# Oral Challenge with Wild-Type *Salmonella* Typhi Induces Distinct Changes in B Cell Subsets in Individuals Who Develop Typhoid Disease

**DOI:** 10.1371/journal.pntd.0004766

**Published:** 2016-06-14

**Authors:** Franklin R. Toapanta, Paula J. Bernal, Stephanie Fresnay, Laurence S. Magder, Thomas C. Darton, Claire Jones, Claire S. Waddington, Christoph J. Blohmke, Brian Angus, Myron M. Levine, Andrew J. Pollard, Marcelo B. Sztein

**Affiliations:** 1 Center for Vaccine Development, University of Maryland School of Medicine, Baltimore, Maryland, United States of America; 2 Department of Medicine, University of Maryland School of Medicine, Baltimore, Maryland, United States of America; 3 Department of Pediatrics, University of Maryland School of Medicine, Baltimore, Maryland, United States of America; 4 Department of Epidemiology and Public Health, University of Maryland School of Medicine, Baltimore, Maryland, United States of America; 5 Oxford Vaccine Group, Department of Paediatrics, University of Oxford and the NIHR Oxford Biomedical Research Centre, Oxford, United Kingdom; 6 Nuffield Department of Medicine, University of Oxford, Oxford, United Kingdom; Massachusetts General Hospital, UNITED STATES

## Abstract

A novel human oral challenge model with wild-type *Salmonella* Typhi (*S*. Typhi) was recently established by the Oxford Vaccine Group. In this model, 10^4^ CFU of *Salmonella* resulted in 65% of participants developing typhoid fever (referred here as typhoid diagnosis -TD-) 6–9 days post-challenge. TD was diagnosed in participants meeting clinical (oral temperature ≥38°C for ≥12h) and/or microbiological (*S*. Typhi bacteremia) endpoints. Changes in B cell subpopulations following *S*. Typhi challenge remain undefined. To address this issue, a subset of volunteers (6 TD and 4 who did not develop TD -NoTD-) was evaluated. Notable changes included reduction in the frequency of B cells (cells/ml) of TD volunteers during disease days and increase in plasmablasts (PB) during the recovery phase (>day 14). Additionally, a portion of PB of TD volunteers showed a significant increase in activation (CD40, CD21) and gut homing (integrin α4β7) molecules. Furthermore, all B_M_ subsets of TD volunteers showed changes induced by *S*. Typhi infections such as a decrease in CD21 in switched memory (Sm) CD27+ and Sm CD27- cells as well as upregulation of CD40 in unswitched memory (Um) and Naïve cells. Furthermore, changes in the signaling profile of some B_M_ subsets were identified after *S*. Typhi-LPS stimulation around time of disease. Notably, naïve cells of TD (compared to NoTD) volunteers showed a higher percentage of cells phosphorylating Akt suggesting enhanced survival of these cells. Interestingly, most these changes were temporally associated with disease onset. This is the first study to describe differences in B cell subsets directly related to clinical outcome following oral challenge with wild-type *S*. Typhi in humans.

## Author Summary

Typhoid fever continues to be a public health problem and novel more effective vaccines are needed. To gain new insights into the host-pathogen interactions, which could aid in novel vaccine design, an improved human oral challenge model with wild-type *Salmonella* Typhi (*S*. Typhi) was recently developed. In this model, 65% of the challenged subjects developed typhoid fever (referred here as typhoid diagnosis -TD-). B cells, whose main function is antibody production, are crucial components of the adaptive immune system. The effects that *S*. Typhi infection has on B cells have not been explored before. Here we demonstrate that *S*. Typhi induces several changes in various B cell subsets of participants that developed typhoid fever. Notable changes included upregulation of activation markers by a B cell subset that actively produces antibodies (plasmablasts). These cells also upregulated markers that guide them to the gut, the main site of *S*. Typhi infection. Various other changes were identified in other B cell subsets (e.g., Sm CD27+, SmCD27- and naïve) including upregulation of activation molecules (e.g., CD40) and downregulation of co-stimulators (e.g., CD21) that might indicate that each subset plays a different role during typhoid disease. Importantly, these changes were identified mainly in volunteers diagnosed with typhoid disease.

## Introduction

*Salmonella enterica* serovar Typhi (*S*. Typhi) is a human-restricted pathogen and the agent responsible for typhoid fever, a disease that continues to be a major global public health problem [1–3]. Due in part to the absence of a suitable animal model, several aspects of the human response to *S*. Typhi infection remain to be explored [4, 5]. A successful human oral infection model of *S*. Typhi, which allowed studying various aspects of the host-pathogen interaction as well as test vaccines and alternative treatment options, was developed forty years at the University of Maryland [4, 6–10]. A new controlled human infection model of *S*. Typhi was recently developed at the Centre for Clinical Vaccinology and Tropical Medicine, University of Oxford (Oxford Vaccine Group). In this new model, participants were challenged with up to 10^4^ CFU of *S*. Typhi (Quailes strain) in a sodium bicarbonate buffered solution. This dose resulted in 65% of participants being diagnosed with typhoid fever (referred here as typhoid diagnosis -TD-) [11].

Immunity to *S*. Typhi is not well understood, though it is believed to be complex involving local and systemic antibody and cell mediated immunity (CMI) components. Until very recently, the principal role of B cells was considered to be antibody production and antigen presentation; however, recent reports have demonstrated that the B cell compartment is quite complex and involves multiple subsets [12–16]. Interestingly, various B memory (B_M_) subsets have been associated with certain diseases and novel functions [12, 17–19]. For example, using one of the most widely accepted classification schemes (IgD/CD27), four B_M_ subsets are defined [12]: (i) naïve [IgD+CD27-], (ii) unswitched memory (Um) [CD27+IgD+], (iii) switched memory CD27+ (Sm CD27+) [CD27+IgD-] and (iv) Sm CD27- [CD27-IgD-]. Among these subsets, the frequency of Sm CD27- cells has been shown to increase in patients with respiratory syncytial virus (RSV) infections [12]. Additionally, Um cells seem to play a crucial role in response to encapsulated pathogens (e.g., *S*. *pneumonia* or *N*. *gonorrhea*) [18, 20, 21]. In the case of *S*. Typhi, there is evidence of the importance of the B cell compartment in protection from disease. For example, the purified Vi polysaccharide administered as a parenteral vaccine, which is a T-independent antigen that activates only B cells, is efficacious in the prevention of typhoid fever; therefore, demonstrating that serum Vi antibodies can mediate protection. Additionally, another typhoid vaccine, Ty21a, elicits serum IgG antibodies against lipopolysaccharide (LPS) O-antigen, which correlates with the level of protection conferred by some, but not other, formulations and immunization schedules [4, 22]. Further evidence comes from studies showing that B cells are able to cross-present *Salmonella* antigens and activate CD8+ T cells, a process that depends on CD4 T cell help [23]. In the rodent model of typhoid (*S*. Typhimurium) more evidence of the role of B cells has been reported using B cell-deficient mice (Igh-6^-/-^ or Igμ^-/-^) [24–26]. For example, B cell-deficient mice that were vaccinated with a live-attenuated strain of *Salmonella* and subsequently challenged with a virulent strain (SL1344) were unable to resist infection [27]. Of note, adoptive transfer of immune serum to vaccinated B cell-deficient mice (Igμ^-/-^) the day previous to challenge (virulent *Salmonella* SL1344) successfully reconstituted their immunity [25]. Moreover, in B cells, Toll-like receptor (TLR) stimulation appears to drive appropriate development of humoral responses, as demonstrated in mice with B cells deficient in MyD88. In these animals, *Salmonella* infections resulted in impaired IgG2b, IgG2c, IgA and IgM responses compared to mice with functional MyD88 [28]. These animals also showed impairment in the development of IFN-γ effector cells mainly due to deficient cytokine production by B cells [29], suggesting a role for B cells in T cell differentiation, which depended on TLR stimulation. Importantly, in human B cells, TLR stimulation (e.g., TLR-2, TLR-5, TLR-7 and TLR-9, but not TLR-4 since human B cells do not express this receptor) has also been suggested as a requirement for effective activation [30]. Other studies are providing insights into the interactions between *Salmonella* and B cells [31]. For example, B cell infection by *S*. Typhimurium was reported and this process depended on antigen-specific BCRs (on the B cell side) and a functional Type-III secretion system (T3SS) (on the bacterium side) [32–34]. Additionally, *S*. Typhimurium is able to modulate ongoing immune responses by facilitating the development of regulatory B cells (immune-suppressive) [35, 36]. Finally, *S*. Typhimurium can induce B cells survival, a process that dependents on inhibition of the inflammasone and that requires the bacteria T3SS SPI-1 [37]. Induction of B cell survival benefits *Salmonella* because the bacteria use the cells as a survival and dissemination niche [33]. Finally, while the existence of human B_M_ cells to *S*. Typhi was suspected for many years, only recently has our group provided the first direct evidence for the presence of *S*. Typhi-specific B_M_ cells (IgA and IgG anti-LPS and -Vi) in volunteers immunized with vaccines for *S*. Typhi [38, 39]. Despite these advances, our knowledge regarding human B cell responses in typhoid fever is still limited. For example, it is unknown whether a specific B cell subset has a predominant function in typhoid disease as described for other pathogens and the changes induced in these cells following immunization and/or infection. Furthermore, whether similar *Salmonella*-B cell interaction as described above for *S*. Typhimurium are operational in humans infected with *S*. Typhi remain to be explored. Evaluation of these phenomena in humans has been impaired since specimens from individuals infected with wild-type (wt) *S*. Typhi are difficult to obtain in field settings. The development of a new human infection model of typhoid fever has provided a unique opportunity to explore important questions about the role of circulating B cells and their various memory subsets in this disease. In the current study we report changes in frequency, activation and migration of various B_M_ subsets in participants with typhoid diagnosis (TD) and those who did not developed disease (NoTD) following wild-type challenge with *S*. Typhi. Furthermore, we explore changes in activation of *S*. Typhi-LPS-specific B_M_ cells and contrast the differences between TD and NoTD volunteers.

## Methods

### Human volunteers, clinical trial description and ethics statement

The specimens (peripheral blood mononuclear cells -PBMC-) used in the current study were collected as part of a clinical trial performed at the University of Oxford (Centre for Clinical Vaccinology and Tropical Medicine) aimed at developing a new human model of ***S***. Typhi infection. The clinical results of this study have already been published [11]. In short, healthy adult (18–60 years-old) individuals without previous history of typhoid vaccination or residence (>6 months) in endemic areas were included in the study. Previous to oral challenge, the volunteers fasted for 90 minutes before ingesting 120 mL/2.1 g NaHCO_3_(aq). The bacteria inocula (*S*. Typhi -Quailes strain- 10^4^ CFU) were prepared in 30 mL/0.53 g NaHCO_3_(aq) which was administered 2 minutes after the volunteers ingested the 120 mL/2.1 g NaHCO_3_(aq). Following oral challenge, the participants were evaluated daily for at least 14 days. During this time, solicited and unsolicited symptoms experienced by the participants as well as oral temperature readings (2 times per day) were recorded. Typhoid fever diagnosis included reaching clinical (temperature ≥ 38°C sustained for ≥ 12 hours) and/or microbiological (blood culture confirmed *S*. Typhi bacteremia) endpoints. Antibiotic treatment (ciprofloxacin, 500 mg twice daily, 14 days) was indicated when (i) typhoid was diagnosed, (ii) unmanageable symptoms were present or (iii) due to clinical necessity. Additionally, all volunteers who did not develop typhoid fever received antibiotic treatment at day 14. Additional follow-up visits were completed at days 21 and 28 days post-challenge. In the current study a subset of individuals (6 TD and 4 NoTD) were evaluated for changes in B cells. These volunteers were selected based on specimen availability at critical time points to evaluate B cell responses.

All volunteers enrolled in the study provided a written informed consent and the procedures were approved by the Oxfordshire Research Ethics Committee A (10/H0604/53). This trial was registered on the UK Clinical Research Network (identifier UKCRN ID 9297). Additionally, in order to optimize flow cytometry panels and other assays, PBMC from healthy adult volunteers recruited from the Baltimore-Washington area and the Center for Vaccine Development (CVD) of the University of Maryland (UMB) were used. These volunteers also provided written informed consent and the procedures approved by the UMB IRB (HCR-HP-00040025).

### Isolation of PBMC

PBMC isolation (density gradient centrifugation) and cell cryopreservation from blood samples of volunteers challenged with *S*. Typhi (Quailes strain) were performed before (day 0) and after (various time points) challenge as previously described [40, 41]. The time points evaluated differed slightly between TD and NoTD volunteers. Days 0 (pre-challenge), 1, 2, 4, 7, 9, 14, 21 and 28 were evaluated in all subjects. Additional samples were collected in TD volunteers, which included the time at which typhoid was diagnosed (6–9 days after challenge [11]) as well as 48 and 96 hours later.

### Staining for flow-cytometry

Cryopreserved PBMC were thawed and allowed to rest overnight (37°C, 5% CO_2_) as previously described [41, 42]. Plating of cells (1x10^6^), staining for viability, bacteria binding (50:1—bacteria:cells ratio [43]), blocking (human IgG -25 μl of a 1 mg/ml solution; mouse IgG -25 μl of a 200 μg/ml solution) and staining of surface targets with monoclonal antibodies were performed as described in detail in [40]. Monoclonal antibodies (mAbs) against the following molecules were used: CD19-ECD (clone J3-119; Beckman Coulter -BC-), CD38-PE-Cy5 (clone LS1298-4-3; BC), CD14-QDot 655 (clone TuK; Invitrogen), CD21-BV711 (clone B-ly4; Becton-Dickinson -BD-), integrin α4β7-Alexa647 (clone ACT-1; Millennium, The Takeda Oncology Co), CD3-Alexa Fluor 700 (clone UCHT1; BD), IgD-FITC (polyclonal goat anti-sera; Southern Biotech), CD27-PE (clone L128; BD), CD40-PE-Cy7 (clone 5C3; BD), IgA-Biotin (polyclonal goat anti-sera; Southern Biotech) and Pacific Orange-Streptavidin (Invitrogen, USA). Finally, stained cells were fixed with 1% PFA in PBS until data collection in a LSRII (BD) instrument.

### Phosphoflow assay

#### Stimulants used

PBMC were stimulated with *S*. Typhi-LPS-QDot655 micelles of nanoparticle size (approx. 30–60 nm) (LPS-nanoparticles) which were generated as previously described [40, 44–46]. Stimulation of PBMC was with approximately 5 μg of LPS (contained in the *S*. Typhi-LPS-nanoparticles). Additionally, as positive and negative stimulation controls H_2_O_2_ (6 mM) (a general phosphatase inhibitor) and 1% BSA (in PBS) were used, respectively.

#### Stimulation and staining

Thawing, staining for viability, stimulation (LPS-nanoparticles, 6 mM H_2_O_2_ and 1% BSA), fixation (80% methanol), blocking (human IgG -25 μl of a 1 mg/ml solution; mouse IgG -25 μl of a 200 μg/ml solution) and staining of surface and intracellular targets were performed as described in detail in [40]. mAbs used included: IgD-FITC (goat polyclonal; SouthernBiotech), CD27-PE (clone L128; BD), pAKT-S473-Ax647 (clone D9E; Cell Signaling Technologies), CD20-PerCP-Cy5.5 (clone H1; BD), p38MAPK-T180/Y182-Pacific Blue (clone 36/p38 (pT180/pY182); BD), Erk1/2-T202/Y204-PE-CF594 (clone 20A; BD), p38MAPK-T180/Y182- PE-CF594 (clone 36/p38 (pT180/pY182), Btk-Y551-Alexa647 (clone BtkY551 & ItkY511; BD) and/or NFκB p65-pS529-PE-Cy7 (clone K10-895.12.50 (pS529; BD). Stained cells were fixed with 1% PFA in PBS until data collection in a LSRII (BD) instrument. Finally, all samples were analyzed using FlowJo (Tree Star, San Francisco, CA) and Cytobank (Palo Alto, CA) software packages.

### Statistical methods

The frequency of the cells (per ml of blood) or percentages of the various B cell subsets in TD and NoTD volunteers before challenge were compared using Mann-Whitney tests. The frequency of cells (cell/ml) was calculated using the number of lymphocytes (per ml of blood) as determined in the white cell counts (WCC) for each volunteer. WCC were performed at various time points after challenge and these data have already been published in [11]. Among those who acquired disease, the disease onset occurred 6–9 days post-challenge (10^4^ CFU); however, since not all volunteers developed typhoid at the same time, the data was grouped in narrow time frames, defined by the exact onset date, to facilitate analysis and interpretation of the data. These time frames included: Around Time of Disease (AroundTD) and After Time of Disease (AfterTD). AroundTD in TD volunteers encompassed the day in which typhoid was diagnosed (TD+0h) until 96 hours post-diagnosis (TD+96h). Importantly, in TD volunteers two extra blood samples were collected at 48 and 96 hours post-diagnosis (TD+48h and TD+96h, respectively). In NoTD volunteers, the AroundTD time frame corresponds to days 7–11 (D7-11). The AfterTD time frame in TD volunteers involves all time points >TD+96h. Meanwhile, AfterTD in NoTD volunteers encompassed all time points >day 11 (>D11) after challenge. We used mixed effects models in order to compare mean values by time period (AroundTD and AfterTD) and group (TD and NoTD), while still accounting for the lack of independence between multiple measures from the same volunteer at the same time period and across time periods. The mixed effects models evaluation included a random effect for subject, fitted by restricted maximum likelihood. We have confirmed that this approach provides valid statistical inference for data sets of this size through various simulation experiments. All hypotheses in the study were evaluated using two-sided tests and p values <0.05 (two-sided) without adjustment for multiple comparisons were considered statistically significant. Finally, in TD volunteers, correlations (Spearman) between populations that demonstrated changes in markers and the fold-increase in antibody titers were evaluated at various time frames (Prism V6, CA).

## Results

### Changes in B cells and plasmablasts (PB) induced by *S*. Typhi infection

**Fig 1:**
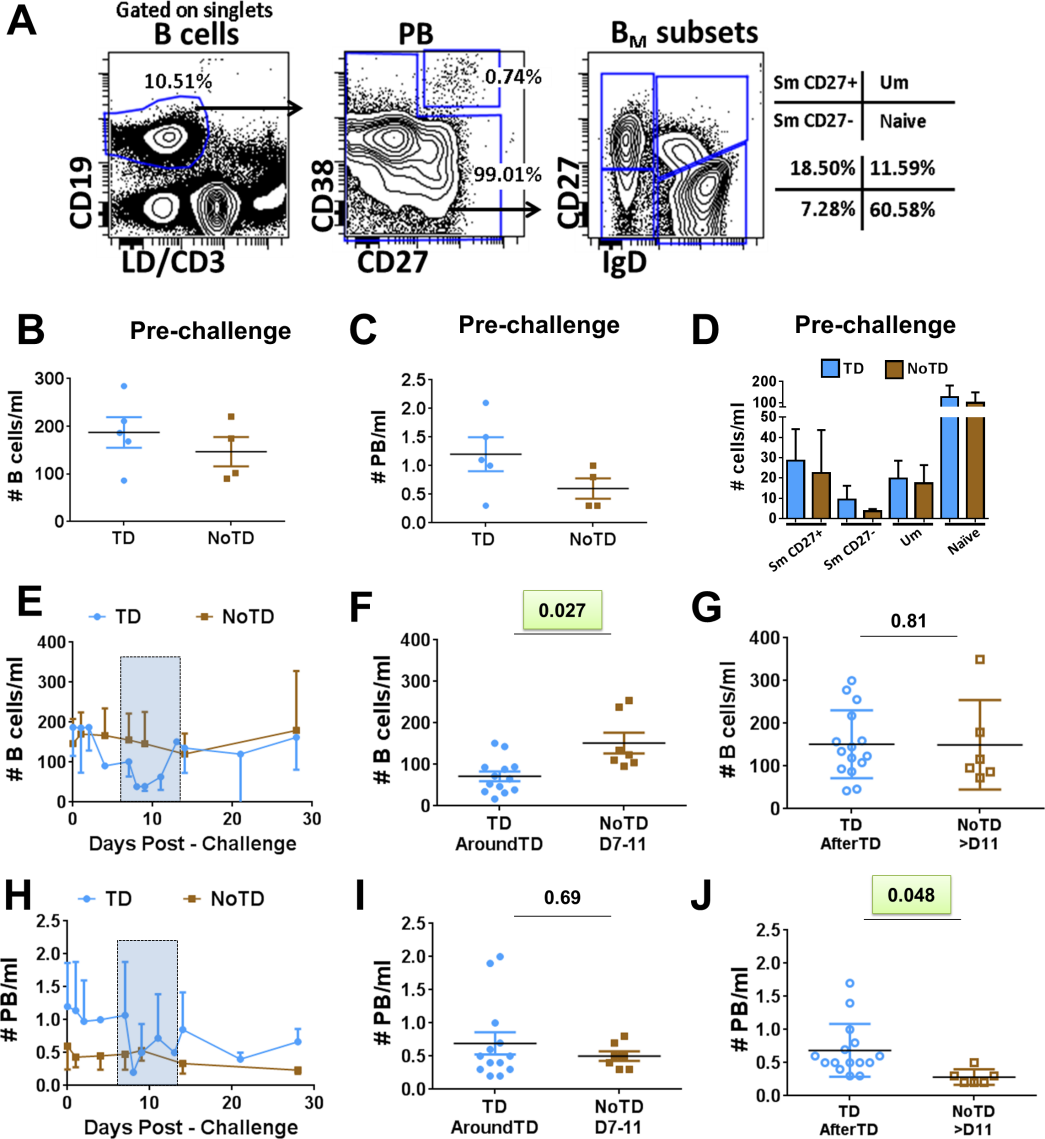
Changes in frequency (cells/ml) of B cells induced by wt *S*. Typhi infection. Panel **A** displays the gating strategy used to identify various B cell subsets. Whole B cells were identified using CD19. Plasmablasts (PB) were identified as B cells (CD19+) expressing high levels of CD27 and CD38. Non-PB cells were classified into various memory B (B_M_) subsets using the IgD/CD27 classification: (**i**) Sm CD27+ (CD27+IgD-), (**ii**) Sm CD27- (CD27-IgD-), (**iii**) Um (CD27+IgD+) and (**iv**) Naïve (CD27-IgD+). The frequency (cells/ml) of whole B cells, PB and B_M_ subsets in TD (blue symbols) and NoTD (brown symbols) volunteers before wild-type challenge is shown in **B**, **C** and **D,** respectively. Shown in panel **E** are the time courses of the frequency of B cells after challenge in TD (solid blue circles) and NoTD (solid brown squares) volunteers. The time frame AroundTD is indicated by the blue rectangle with dotted lines. AroundTD in TD volunteers include the day in which typhoid was diagnosed (TD+0h) and two extra time points, 48h and 96h post-diagnosis (TD+48h and TD+96h, respectively), in which blood samples were collected. Shown in **F** are the data of B cells from TD (solid blue circles) and NoTD (solid brown squares) volunteers in the AroundTD time frame. Shown in **G** are the data of B cells from TD (open blue circles) and NoTD (open brown squares) in the AfterTD time frame (days 14, 21 and 28 for TD and NoTD volunteers). Time course, AroundTD and AfterTD data of the frequency of plasmablasts (PB) is shown in **H**, **I** and **J**, respectively. Shown is the P value from the mixed effects model analysis. Significant differences are highlighted in light green. Mean ± SD are presented in all graphs.

**Fig 2:**
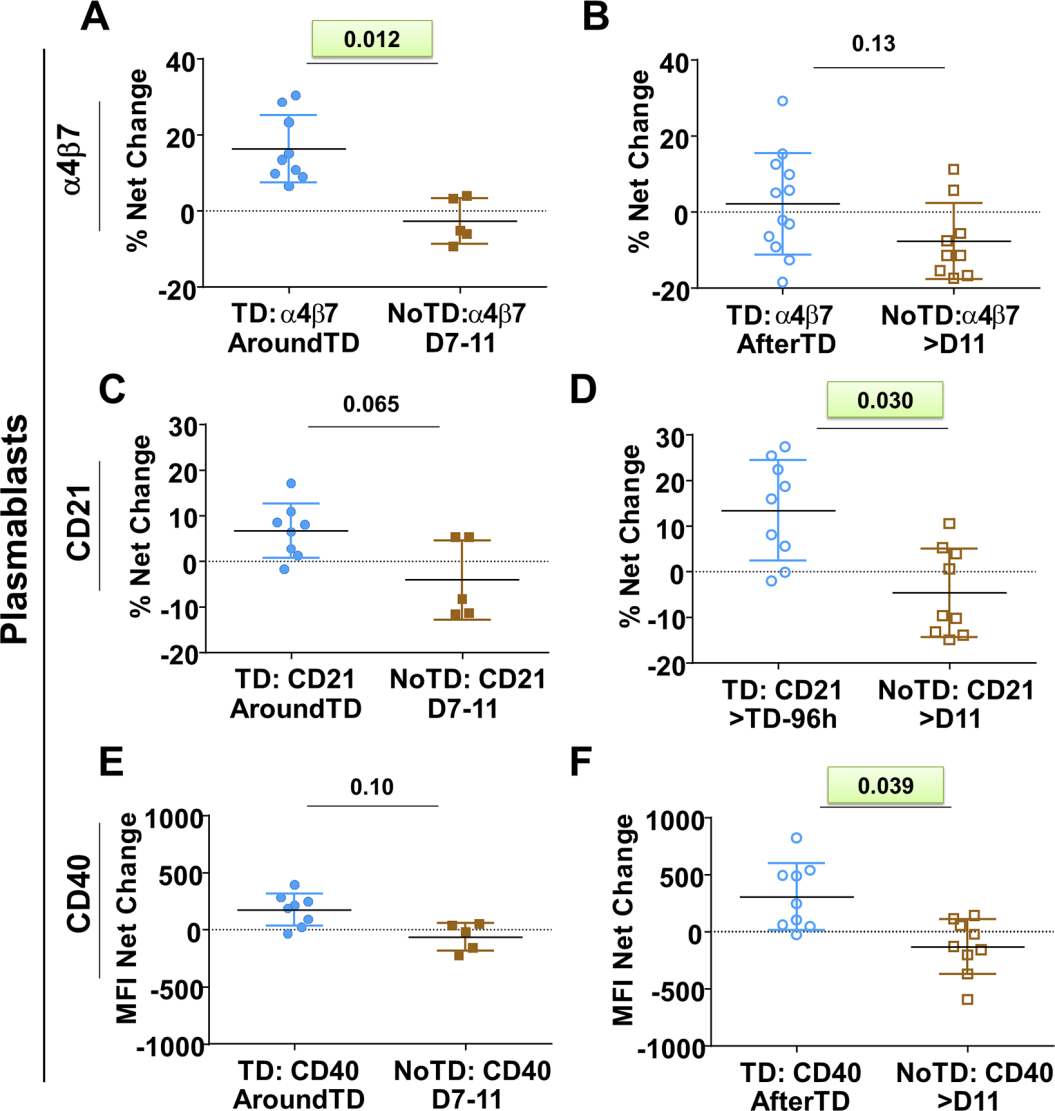
Changes in expression of surface molecules in PB induced by wt *S*. Typhi infection. Shown in panels **A**, **C** and **E** are the expression of integrin α4β7, CD21 and CD40, respectively, from TD (solid blue circles) and NoTD (solid brown squares) volunteers in the AroundTD time frame. Panels **B**, **D** and **F** show the expression of integrin α4β7, CD21 and CD40, respectively, from TD (open blue circles) and NoTD (open brown squares) in the AfterTD time frame. The P value from the mixed effects model analysis is shown. Significant differences are highlighted in light green. Mean ± SD are presented in all graphs.

B cells were identified using CD19 (CD19^+^ CD3^-^) (**Fig 1A**). Within B cells, plasmablasts (PB) were identified as cells expressing high levels of CD27 and CD38 (CD27^high^ and CD38^high^). Non-PB cells were further categorized into various B memory (B_M_) subsets using the IgD/CD27 classification scheme as proposed by Sanz et al [12], which delineates four subpopulations: (i) Sm CD27+ [CD27+IgD-], (ii) Sm CD27- [CD27-IgD-], (iii) Um [CD27+IgD+] and (iv) naïve [CD27-IgD+] cells. No significant differences were observed in the frequency of all B cells (B cells/ml) (**Fig 1B**), PB (PB/ml) (**Fig 1C**) or the various B_M_ subsets (cells/ml) (**Fig 1D**) before *S*. Typhi challenge between participants who were diagnosed with typhoid (TD) and those who were not (NoTD). We next assessed changes in the number of whole B cells (cell/ml) after challenge (**Fig 1E–1G**). A significant reduction in the number of B cells in TD (compared to NoTD) volunteers was observed AroundTD (**Fig 1F**). The reduction of these cells was transitory as no differences between TD and NoTD volunteers was observed AfterTD (**Fig 1G**). Of note, PB did not show statistically significant differences in the frequencies between TD and NoTD AroundTD (**Fig 1H and 1I**). However, an increase in frequency of these cells was noted AfterTD (**Fig 1J**). We then evaluated various molecules on PB including gut homing (integrin α4β7), activation (CD40 and CD21) and class switching markers (IgA) as well as the ability of these cells to bind *S*. Typhi (**Fig 2** and **S1 Fig**). These data are reported as net differences (percentages or MFI) AroundTD or AfterTD relative to day 0. TD volunteers showed a significant upregulation of integrin α4β7, during the AroundTD time frame, compared to NoTD volunteers (**Figs 2A, S1A and S1B)**. This homing marker returned to pre-challenge levels AfterTD (**Fig 2B**). An up-regulation of CD21 and CD40 was also noted (**Fig 2C–2F** and **S1D–S1F Fig**) in TD volunteers and the increase was significant in the AfterTD time frame (**Fig 2D** and **2F**). Moreover, the upregulation of CD21 by PB (AfterTD) correlated with the increase of anti-flagella IgG antibody titers, but not anti-LPS or anti-Vi, AfterTD (**S1I Fig**) [11]. Examples of the differences between pre-challenge and post-challenge in TD volunteers as well as the time courses of the changes in these markers are included in **S1 Fig** Finally, no significant differences between TD and NoTD volunteers were noted in the expression of IgA or the ability of PB to bind *S*. Typhi (**S1 Fig**).

### Changes in B_M_ subsets following wt *S*. Typhi infection

**Fig 3:**
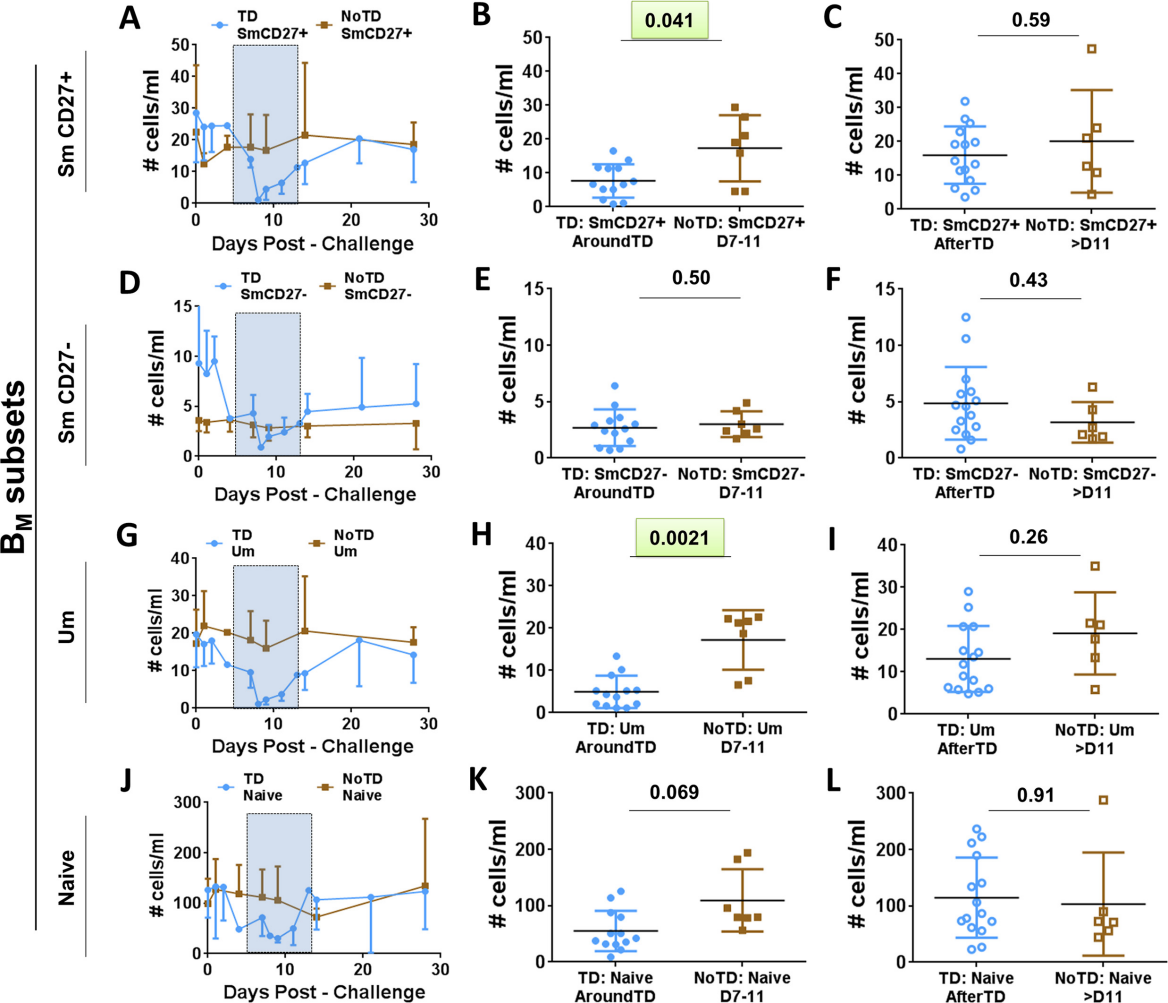
Changes in frequency (cells/ml) of B_M_ subsets induced by wt *S*. Typhi infection. B_M_ subsets (IgD/CD27 classification) were evaluated for changes in frequency (cells/ml) elicited by exposure to wt *S*. Typhi. Panels **A**, **D, G** and **J** display the time courses of the changes in Sm CD27+, Sm CD27-, Um and Naïve cells, respectively, in TD and NoTD volunteers (blue and brown simbols, respectively). The AroundTD time frame is indicated in panels **A, D, G** and **J** by the blue rectangles with dotted lines. Panels **B**, **E, H** and **K** shown changes in Sm CD27+, Sm CD27-, Um and Naïve subsets, respectively, from TD (solid blue circles) and NoTD (solid brown squares) volunteers in the AroundTD time frame. Panels **C**, **F, I** and L, show changes in Sm CD27+, Sm CD276-, Um and Naïve subsets, respectively, from TD (open blue circles) and NoTD (open brown squares) in the AfterTD time frame. The P value from the mixed effects model analysis is shown. Significant differences are highlighted in light green. Mean ± SD are presented in all graphs.

**Fig 4:**
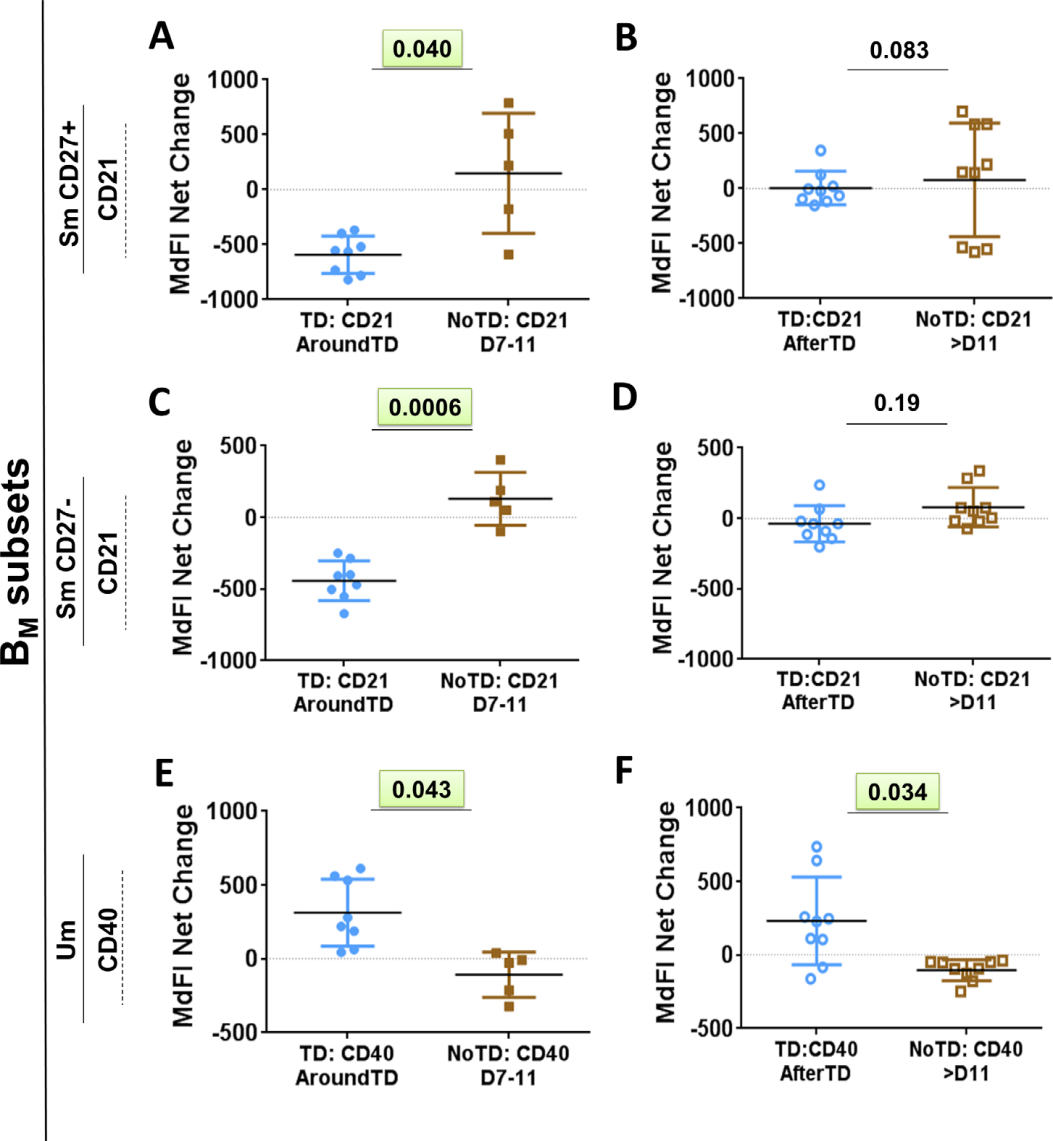
Changes in surface molecules of Sm CD27+, Sm CD27- and Um subsets induced by wt *S*. Typhi infection. Shown in panels **A**, **C** and **E** are the expression of CD21 and CD40 from TD (solid blue circles) and NoTD (solid brown squares) volunteers in the AroundTD time frame. Panels **B**, **D** and **F** show the expression of CD21 and CD40 from TD (open blue circles) and NoTD (open brown squares) volunteers in the AfterTD time frame. The P value from the mixed effects model analysis is shown. Significant differences are highlighted in light green. Mean ± SD are presented in all graphs.

**Fig 5:**
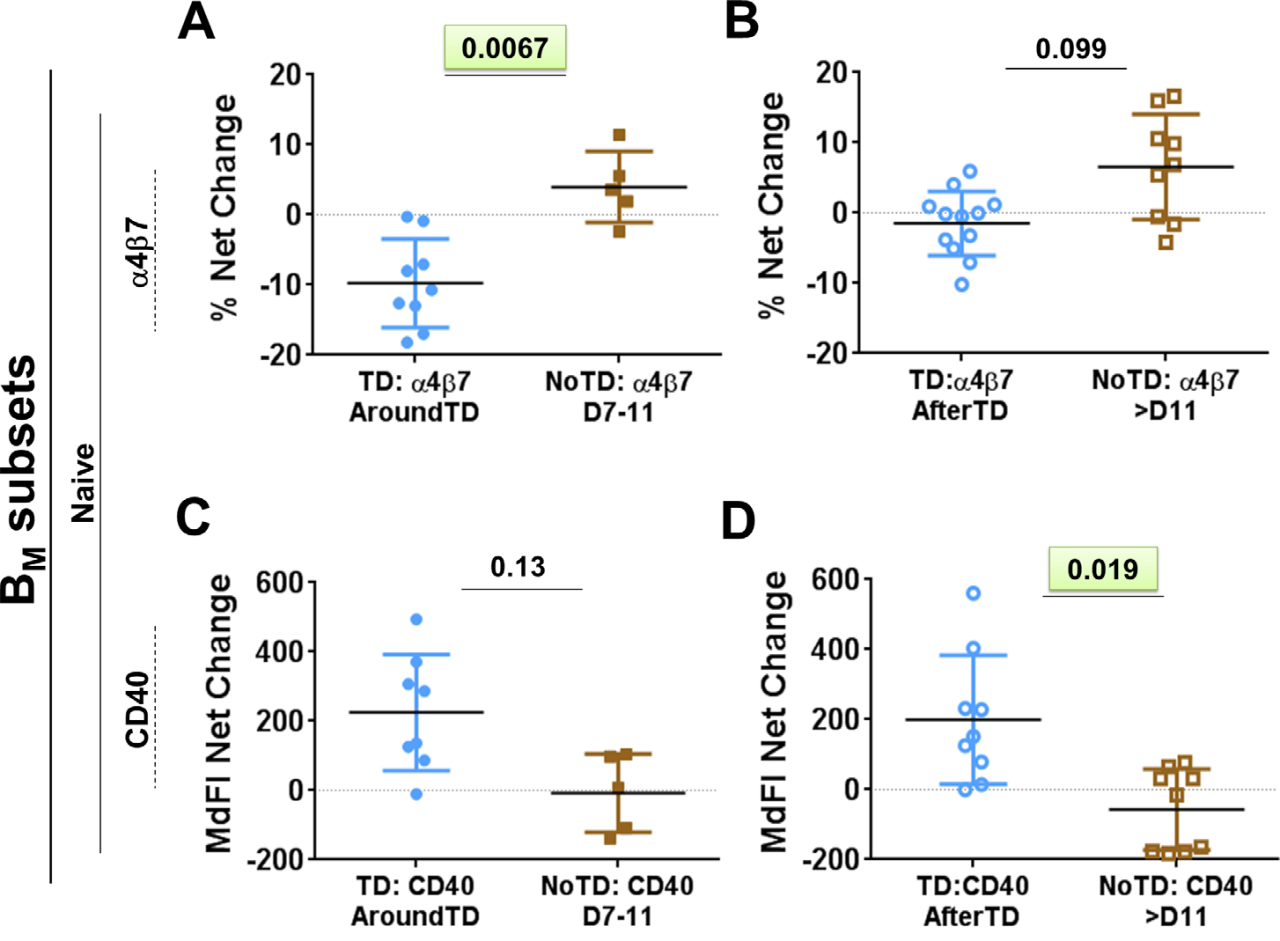
Changes in surface molecules of Naive cells induced by wt *S*. Typhi infection. Shown in panels **A** and **C** are the expression of integrin α4β7 and CD40 from TD (solid blue circles) and NoTD (solid brown squares) volunteers in the AroundTD time frame. Panels **B** and **D** show the expression of integrin α4β7 and CD40 from TD (open blue circles) and NoTD (open brown squares) volunteers in the AfterTD time frame. The P value from the mixed effects model analysis is shown. Significant differences are highlighted in light green. Mean ± SD are presented in all graphs.

Evaluation of the frequency (cells/ml) of the B_M_ subsets (IgD/CD27 classification) between TD and NoTD volunteers before challenge showed no significant differences among these groups (**Fig 1D**). Changes in the frequency (cells/ml) of the various B_M_ subsets after challenge (**Fig 3**) demonstrated that SmCD27+ and Um cells of TD volunteers decreased significantly AroundTD, compared to NoTD volunteers (**Fig 3A, 3B, 3G and 3H**). However, the decrease was transitory, since the frequency of the cells returned to pre-challenge levels by day 21 (**Fig 3C and 3I**). Changes in frequency were not evident in Sm CD27- or naïve cells (**Fig 3D, 3E, 3J and 3K**). When the data was analyzed as net percentage changes, only Um cells of TD volunteers (compared to NoTD) showed a significant decrease in AroundTD time frame (**S2A–S2D Fig**). Next, we evaluated changes in the expression of various molecules (e.g., CD21, CD40, integrin α4β7 and IgA, as well as *S*. Typhi binding) within each B_M_ subset (reported as net changes relative to day 0) (**Figs 4, 5**and **S2**). Interestingly, despite that only SmCD27+ and Um cells showed reduction in their frequency AroundTD, all B_M_ subsets showed significant changes in some of the evaluated markers and most of them were AroundTD. However, in each B_M_ subset the marker(s) altered were different. TD volunteers downregulated CD21 in Sm CD27+ and Sm CD27- cells AroundTD, compared to NoTD volunteers (**Fig 4A–4D** and **S2E–S2H Fig**). In these volunteers, CD21 returned to pre-challenge levels AfterTD (**Fig 4B–4D** and **S2E–S2H Fig**). In contrast, Um cells showed a significant up-regulation of CD40 AroundTD and AfterTD (compared to NoTD) (**Fig 4E–4F** and **S2I and S2J Fig**). Naïve cells of TD volunteers showed a significant down-regulation of integrin α4β7 AroundTD (compared to NoTD) and this marker returned to pre-challenge levels around day 21 (**Figs 5A, 5B, S2K and S2L**). In naïve cells, up-regulation of CD40 was also present. This marker started to increase AroundTD (**S3A and S3B Fig**) in TD volunteers, but statistically significant differences were demonstrated only AfterTD (**Fig 5D**). The levels of IgA remained unchanged in Sm CD27+ and Sm CD27- cells compared to pre-challenge and no differences between TD and NoTD volunteers were identified in any time frame (**S3C** and **S3D Fig**). Similarly, no B_M_ subset showed significant changes in the binding to *S*. Typhi (**S3E–S3H Fig**). A table summarizing all the markers evaluated in the various B subsets is displayed in **S4 Fig**.

### Identification of antigen-specific B cells by *S*. Typhi-LPS micelles

**Fig 6:**
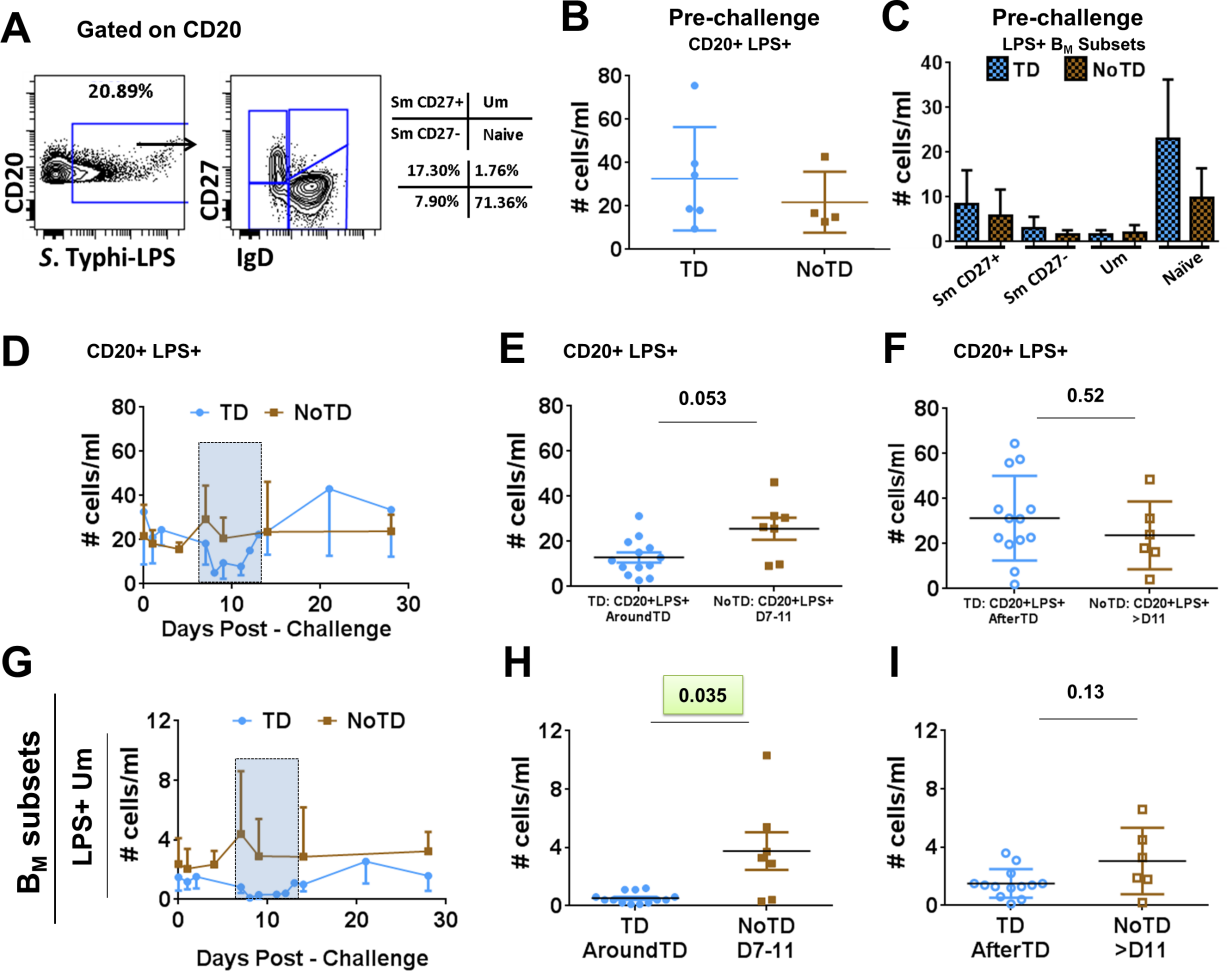
Identification of *S*. Typhi-LPS-specific B cells. Panels **A** shows the gating strategy used to identify *S*. Typhi-LPS-specific B cells using LPS-nanoparticles and the different B_M_ subsets within the *S*. Typhi-LPS specific cells in a representative volunteer. Panels B and C show the frequency (cells/ml) of all *S*. Typhi-LPS-specific B cells, as well as within the B_M_ subsets, in TD (blue symbols) and NoTD (brown symbols) volunteers before wild-type challenge. Panels **D, E** and **F** show the time course, AroundTD and AfterTD, respectively, of the frequency (cells/ml) of *S*. Typhi-LPS-specific B cells in TD (blue symbols) and NoTD (brown symbols) volunteers. Shown in panels **G, H** and **I** are the time course, AroundTD and AfterTD, respectively, of the frequency (cells/ml) of *S*. Typhi-LPS-specific Um (CD20+ LPS+ IgD+ CD27+) cells in TD (blue symbols) and NoTD (brown symbols) volunteers. The P value from the mixed effects model analysis is shown. Significant differences are highlighted in light green. Mean ± SD are presented in all graphs.

**Fig 7:**
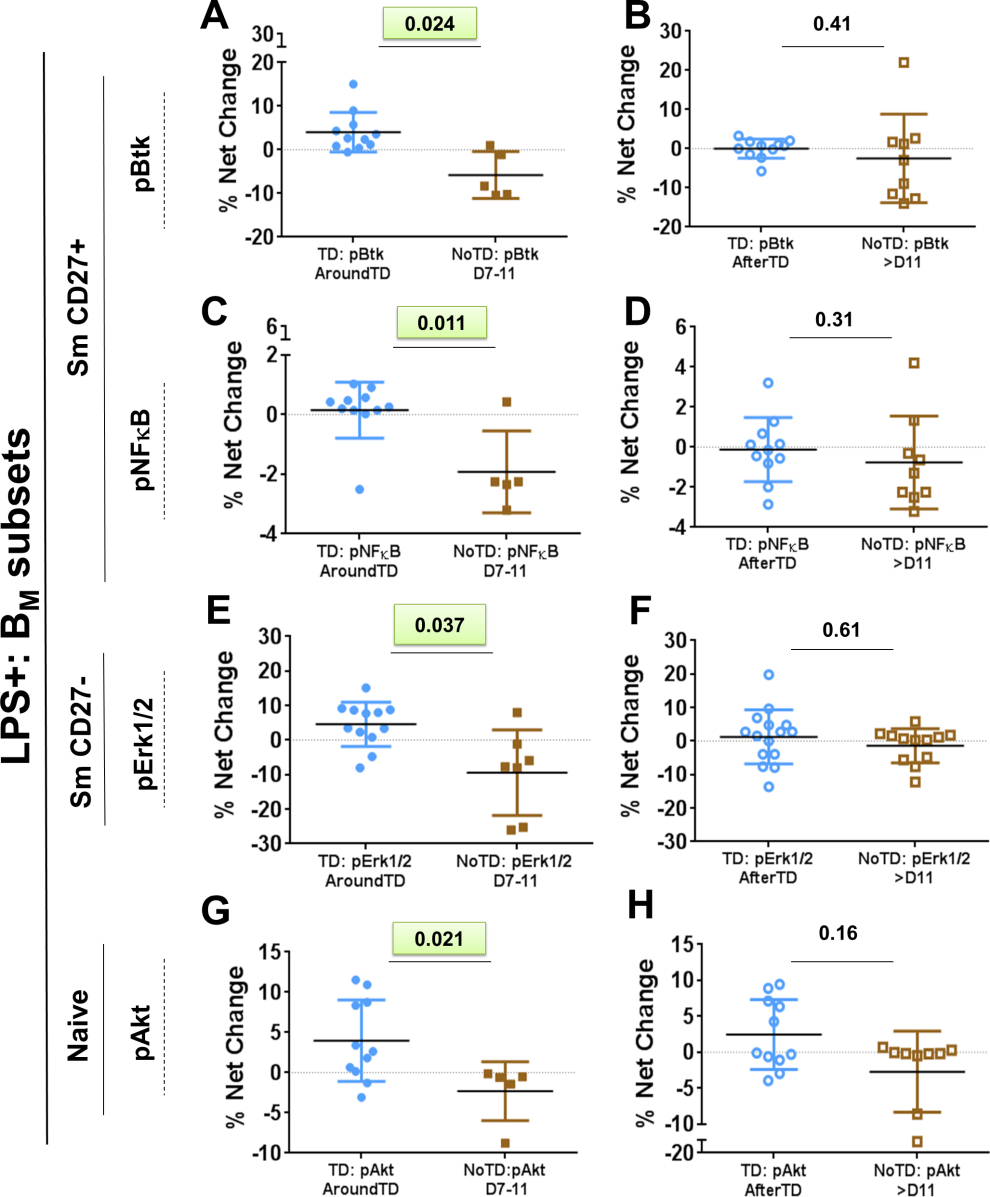
Phosphorylation of diverse signaling molecules in B_M_ subsets following wt *S*. Typhi challenge. The percentages of *S*. Typhi-LPS-specific B cells phosphorylating specific signaling proteins (e.g., Btk, NFkB, Erk1/2 and Akt) were evaluated in each B_M_ subset. Phosphorylation was evaluated after stimulation with *S*. Typhi-LPS-nanoparticles. Panels **A** and **B** show the % net changes of Btk phosphorylation (pBtk) in AroundTD and AfterTD, respectively, of Sm CD27+ cells in TD (blue symbols) and NoTD (brown symbols) volunteers. Panels **C** and **D** show the % net changes of NFκB phosphorylation (pNFκB) in AroundTD and AfterTD, respectively, in Sm CD27+ cells in TD (blue symbols) and NoTD (brown symbols) volunteers. Panels **E** and **F** show the % net changes of Erk1/2 phosphorylation (pErk1/2) AroundTD and AfterTD, respectively, in Sm CD27- cells in TD (blue symbols) and NoTD (brown symbols) volunteers. Panels **G** and **H** show the % net changes of Akt phosphorylation (pAkt) AroundTD and AfterTD, respectively, in naïve cells in TD (blue symbols) and NoTD (brown symbols) volunteers. The P value from the mixed effects model analysis is shown. Significant differences are highlighted in light green. Mean ± SD are presented in all graphs.

LPS is one of the most abundant antigens on the surface of *S*. Typhi and to which anti-O-LPS antibodies are directed. Additionally, LPS-specific memory B cells have recently been reported [38, 39]. To identify and evaluate *S*. Typhi-LPS-specific B cells, *S*. Typhi-LPS micelles of nanoparticle size (LPS-nanoparticles) were used [40]. These LPS-nanoparticles contain fluorescent Quantum dot (Qdot655) cores to allow identification of antigen-specific cells. Furthermore, LPS-nanoparticles are used as stimulants to determine if the receptor(s) interacting with this reagent induce activation of signaling pathways. Since human B cells lack TLR4 and CD14, these receptors cannot induce signaling. However, LPS-specific memory B cells have been reported suggesting that clones specific for this molecule are developed upon infection or vaccination and therefore, the B cell receptor (BCR), which drives the initial clonal selection, is the most likely candidate to interact with LPS. Thus, the main objective of these experiments was to determine changes in the intracellular signaling pathways of B cells induced by *S*. Typhi infection and identify whether differences were present between volunteers who developed disease (TD), and those who did not (NoTD). PBMC were stimulated with LPS-nanoparticles (~5–10 ug; 10 min; 37°C) and then fixed for analysis by phospho-flow. *S*. Typhi-LPS-specific B cells were identified and the distribution of these cells within the different B_M_ subsets was evaluated (**Fig 6A**). No differences in the frequency (cells/ml) of *S*. Typhi-LPS-specific B cells or within the B_M_ subsets between TD and NoTD volunteers before challenge (day 0) were identified (**Fig 6B and 6C**) (**S5A Fig** display gating examples of *S*. Typhi-LPS-specific B cells from TD volunteers on day 0. Additionally, **S5B Fig** and **S5C Fig** show percentage comparison between TD and NoTD groups at day 0 and the percentage distribution in the different B_M_ subsets). Furthermore, after challenge, no differences in the frequency of *S*. Typhi-LPS-specific B cells between TD and NoTD volunteers was identified (**Fig 6D–6F**). Subsequently, we examined changes in the frequency of B_M_ subsets within CD20+ LPS+ B cells. Only Um (CD20+ LPS+ IgD+ CD27+) cells of TD volunteers (compared to NoTD) showed a significant decrease in their frequency AroundTD (**Fig 6G and 6H**). The Um cell frequency was back to pre-challenge levels AfterTD (**Fig 6G–6I**). The other B_M_ subsets did not show significant changes (TD vs. NoTD) in their frequencies (**S5D** and **S5F Fig**). We next evaluated changes (net percentage relative to day 0) in the B_M_ subsets phosphorylating signaling proteins associated with the BCR after challenge. The results were compared between TD and NoTD volunteers (**Fig 7**). The BCR-associated signaling proteins evaluated included: Spleen tyrosine kinase (Syk); Bruton’s tyrosine kinase (Btk), Protein kinase B (Akt), p38 mitogen-activated protein kinase (p38MAPK), extracellular-signal-regulated kinase (Erk) 1/2 and nuclear factor kappa-light-chain-enhancer of activated B cells (NFκB). **S6A Fig** shows examples of the changes in signaling (pAkt) induced by *S*. Typhi-LPS micelle stimulation in naïve (CD20+ LPS+ IgD+ CD27-) cells in 3 volunteers. The examples show the percentage of cells phosphorylating Akt pre-challenge (day 0) and post-challenge (TD+48h or TD+96h). The gates were set up based on cells stimulated with media only (unstimulated control) and the data was reported as percentage differential (% Net changes). Significant differences in the net percentage of cells phosphorylating signaling proteins were identified in TD volunteers (compared to NoTD volunteers) and the changes were concentrated in the AroundTD time frame. These changes were evident in Sm CD27+ (CD20+ LPS+ IgD- CD27+), Sm CD27- (CD20+ LPS+ IgD- CD27-) and naïve (CD20+ LPS+ IgD+ CD27-) cells (**Fig 7**and **S6B–S6E Fig**). In Sm CD27+ cells of TD volunteers, the percentage of cell phosphorylating Btk and NFκB was higher than NoTD volunteers AroundTD (**Fig 7A and 7C**). Sm CD27- cells (from TD volunteers) showed a significantly higher percentage of cells phosphorylating Erk1/2 (**Fig 7E**). Finally, a significant higher percentage of naïve cells phosphorylating Akt was also noted (**Fig 7G**). **S6B–S6E Fig** shows the time course of the changes induced by *S*. Typhi infection in B cells signaling proteins where significant differences were identified. The table shown in **S6F Fig** summarizes the results of phospho-proteins evaluated in all the B_M_ subsets.

## Discussion

Because *S*. Typhi is a human restricted pathogen and no animal model is capable of reproducing all the clinical aspects of this disease, studies in humans are necessary to understand pathogen-human host immune interactions. These studies ultimately hold the potential to aid in the design of novel vaccines. The specimens used in the current study were collected as part of a clinical study aimed at developing a new human challenge model to study typhoid disease [11]. B cells are part of the adaptive immune system which, in addition to being responsible for antibody production, perform other important functions including antigen presentation and cytokine production. Recent advances in B cell biology have shown that the B cell compartment is composed of multiple subsets whose functions are not clearly understood, particularly in the context of infectious diseases. In the current study we aimed at studying changes in the frequency, activation, and signaling induced by *S*. Typhi in peripheral blood B cells of volunteers challenged with 10^4^ CFU of wt *S*. Typhi (Quailes strain). Importantly, by comparing the results between TD and NoTD volunteers we aimed at furthering our understanding of the role of this important component of the human adaptive immune response when first encountering *S*. Typhi, and their potential to impact the development of disease as well as anamnestic responses.

No differences in the frequency (cells/ml) whole B cells (CD19^+^ CD3^-^) or plasmablasts (PB) (CD3^-^ CD19^+^ CD27^high^ CD38^high^) were identified before wild-type challenge, suggesting that it is not possible to predict which volunteers will develop disease by simply looking at the frequency of these cells in circulation (**Fig 1B and 1C)**. Of note, after challenge the frequency of whole B cells (**Fig 1E–1G**) was reduced in volunteers that developed disease (TD), mimicking the results reported in the parent study (decrease of whole cell counts (WCC) and lymphocytes) [11]. We further evaluated the effects of typhoid fever in various B cell subsets. Interestingly, plasmablasts (PB), a B cell subset that produces antibodies early after infection, did not show a reduction in the frequency AroundTD in TD volunteers, but an increase and AfterTD (**Fig 1J**). This increase in frequency was accompanied by upregulation of CD21 and CD40 (**Fig 2D and 2F**), which are co-activator and activator markers, respectively. This suggests that these cells are activated and could be the responsible for giving rise to plasma cells and memory cells [47]. This is further supported by the positive correlation between CD21 upregulation by PB and the increase in anti-flagella antibody titers (**S1I Fig**). Of interest, no correlation was identified with anti-LPS antibody titers.

Integrin α4β7-expressing B and T cells identified in peripheral blood are re-circulating cells that left Peyer’s patches (PP) and/or mesenteric lymph nodes (MLN) upon being primed by intestinal DC [48–51]. Upregulation of integrin α4β7 AroundTD by PB (**Fig 2A**) suggest that a proportion of these cells are getting ready to migrate to the gut, which is not surprising since it is the primary site of infection. This upregulation of integrin α4β7 and antibody production by PB is in agreement with oral vaccination/infection to other enteric diseases such as shigellosis [52].

Similar to observations with whole B cells and PB, no differences were noted in the frequency (cells/ml) of the B_M_ subsets before challenge between TD and NoTD volunteers (**Fig 1D**). However, following wild-type challenge, Sm CD27+ and Um cells showed a significant reduction in their frequency AroundTD in TD volunteers (compared to NoTD) (**Fig 3B, 3H and 3K**), which suggest that some B cell subsets are more affected than other by typhoid fever. Further evidence of the changes induced in B cells by typhoid came from the analysis of activation and homing markers. CD40 was significantly upregulated in a significant proportion of the naïve subset (**Figs 4E–4F** and **5D**) showing that these cells were activated. Considering that naïve B cells give rise to all the other subsets, including PB, plasma cells and memory B cells, it was not surprising to observe that these cells were activated. Of note, naïve B cells also showed downregulation of integrin α4β7 (AroundTD; **Fig 5A**), which is intriguing since it was expected that these cells would migrate to the gut. These data might indicate that naïve B cells first migrate to other secondary organ(s) (currently undefined, but different from the gut) where they differentiate further (e.g., PB) and acquire the expression of integrin α4β7. Alternatively, these data might suggest that different B subsets use different gut homing receptors, for example naïve cells might use CCR9 or CD103 to reach the gut and once these cells commit to another lineage (e.g., PB) integrin α4β7 is upregulated. Yet another possibility is that naïve cells have already migrated to the gut using the integrin α4β7 receptor, resulting in lower levels of circulating integrin α4β7+ B naïve cells. Future studies will be directed to further explore these possibilities.

Um cells have been described to produce high-affinity IgM in the early phase of infections and the equivalent of B1 murine cells [53, 54]. However, the exact origin and function of these cells in humans remains controversial. Here, we demonstrate that these cells play a role in typhoid fever, since despite that their frequency is reduced AroundTD (Fig 3H), some of these cells show signs of activation (**Fig 4E–4F**). Their role in typhoid and other enteric infections remains to be further explored.

CD21 works in conjunction with CD19 (CD19/CD21 complex) to enhance BCR signaling, leading to B cell activation. Downregulation of CD21 by Sm CD27+ and Sm CD27- cells AroundTD in TD volunteers (**Fig 4A and 4C**) might represent a physiological process during the development of a normal B cell response, particularly in its early stages. We observed that CD21 was upregulated in PB (**Fig 2D–2F**), which likely represent antigen-specific cells already producing antibodies and which require activation to continue their developmental process. In contrast, Sm CD27+ and Sm CD27- cells might contain memory B cells with affinity for antigens other than *S*. Typhi LPS. In the case of any infectious disease, the immune response can lead to an environment (e.g., cytokine milieu) that facilitates the activation of innate and antigen specific (adaptive) cells. In the case of B cells, downregulation of co-activators in cells that have not encountered an antigen specific for the invading microorganism might represent a mechanism to limit their activation. To our knowledge, CD21 downregulation has been reported in some autoimmune diseases and these cells are characterized by being anergic [55, 56]. Therefore, these B cells (not *S*. Typhi-specific) might be refractory to activation.

One of the most abundant molecules on the surface of *S*. Typhi and to which antibodies are directed is LPS. LPS is a well-recognized mouse, but not human, B-cell mitogen. This is due to the fact that unlike mouse B cells, human B cells express neither TLR4 nor CD14. However, in humans exposed to *S*. Typhi, anti-LPS-O antibodies are detected and used as an indicator of successful immunization or challenge. This suggests that B cell clones specific for this antigen are generated during the development of immune responses to *S*. Typhi. Considering that clonal selection for B cells occurs through interaction of the antigen with the BCR, the presence of anti-LPS antibodies indicate that LPS was recognized by B cells that developed into antibody producing clones. Therefore, B cell clones that recognize *S*. Typhi-LPS though the BCR allow the identification of changes in signaling profiles elicited by typhoid fever. To this end, we used LPS-nanoparticles (*S*. Typhi-LPS), which contain a quantum dot (QDot 655) core, as stimulants and evaluated the BCR associated signaling pathways induced within the different B_M_ subsets that interacted with the LPS-nanoparticle [40]. Importantly, LPS is a very “sticky” molecule and its “sticky” nature is thought to be dependent on ionic interaction [57]. Therefore, besides the BCR (specific interaction), it is likely that LPS interacts non-specifically with other molecules, especially if they have a net positive charge (LPS has a net negative charge). As expected, due to the large repetitive nature of LPS and its “stickiness”, a relatively high percentage of B cells interacted with the LPS-nanoparticles (high background) (**Fig 6A–6C**). This is evidenced in pre-challenge samples (day 0) where LPS interaction of B cells raged from ~10–25% (**S5A Fig**), despite the fact that these volunteers did not exhibit anti-*S*. Typhi antibodies (anti-LPS, anti-Vi or anti-flagellin) before challenge as previously reported [11]. We expected that only a small percentage of cells interacting with LPS will actually respond to the stimuli (specific interaction), which in our assay was determined by phosphorylation of signaling proteins. To identify the differential in activation of BCR signaling pathways induced by typhoid, each sample was corrected for background using the phosphorylation on each protein induced in day 0. Following wild-type *S*. Typhi challenge, naïve B cells from TD volunteers showed a significant difference (compared to NoTD) in the net percentage of cells phosphorylating Akt AroundTD (**Fig 7G**). Akt is part of the survival pathway and this suggests that naïve B cells of TD volunteers that interact with the LPS-nanoparticles are more resistant to apoptosis. These data complemented the observations that naïve B cells were activated (CD40 upregulation–**Fig 5D)**. Interestingly, we also noticed differences in the Sm CD27+ population, in which NFκB and Btk phosphorylation showed differences between TD and NoTD volunteers AroundTD (**Fig 7A and 7C**). Sm CD27- B cells from TD volunteers also showed differential in the phosphorylation of Erk1/2 (compared to NoTD). Whether either one of these pathways leads to the downregulation of CD21, as identified in the Sm CD27+ and Sm CD27- AroundTD (**Fig 4A and 4C**) remains to be explored. Also, whether activation of these signaling proteins indicate that these cells are memory cells, will be evaluated in future studies. We acknowledge, however, that since we evaluated phosphorylation at a single time point (10 minutes stimulation), we might have captured only a fraction of the changes that these cells are undergoing. Future studies will be directed to evaluate in more detail the kinetics of the signaling pathways associated with the BCR activation in TD and NoTD volunteers.

In summary, the changes induced by *S*. Typhi infection in volunteers that developed disease (TD) were analyzed in various B cell subpopulations. The cells that showed the most important changes were PB which exhibited significant increases in various key activation and gut homing molecules. Naïve B cells showed activation and enhanced survival, which are likely related to their differentiation into PB as well as plasma cells and possibly long term memory B cells. Interestingly, changes in Sm CD27+ and Sm CD27- cells were also identified including downregulating CD21 as a possible mechanism to avoid non-specific activation. All these changes were present only in TD volunteers, suggesting that these were induced by exposure to wt *S*. Typhi and are in response to the development of disease. To our knowledge this is the first study to describe these phenomena on human B cells of volunteers that developed typhoid disease after wild-type challenge. The changes that other *S*. Typhi antigens (e.g., flagella, Vi) induce in the different B_M_ subsets will be addressed in future studies.

## Supporting Information

S1 Fig Changes in PB induced by wt *S*. Typhi infection. Panels **A** and **B** show examples of the changes in the expression of integrin α4β7 and the time course of the net change (percentage) of this molecule in PBs. TD and NoTD volunteers are indicated by the blue and brown symbols, respectively. Panels **C** and **D** show examples of the changes in the expression of CD21 and the time course of the net change (percentage) of this molecule in PBs. Panels **E** and **F** show examples of the changes in the expression of CD40 and the time course of the net change (MFI) of this molecule in PBs. Graphs **A**, **C** and **E** show the data before challenge and peak increases after-challenge. In Panel **E** the bar color bar indicates the net MFI change over pre-challenge. Also sown are the raw MFI values. Panels **G** and **H** show the complete time courses of IgA expression and *S*. Typhi binding in PB in TD (blue symbols) and NoTD (brown symbols) volunteers. AroundTD is indicated by the blue rectangles with dotted lines in panels **B, D, F, G,** and **H**. Panels **B, D, F, G,** and **H** display Mean ± SD. Panel **I** displays the Spearman correlation results between PB CD21+ cells (% net change over day 0; AfterTD time frame) and the anti-flagellin IgG antibody titer (fold-increase over day 0; After TD time frame) in TD volunteers. The AfterTD time frame for the antibody titers included data from days 14, 28 and 60.
(PDF)

S2 Fig Changes in B_M_ subsets induced by wt *S*. Typhi infection (part 1). Panels **A-D** show the time course of the net changes (percentage) of the various B_M_ populations. The p value (mixed effects model) for significant differences between TD and NoTD volunteers at AroundTD is indicated in the green rectangle. Panels **E** and **F** show examples of the changes in CD21 expression in Sm CD27+ (TD volunteer) and the time course of the net change (percentage) of this molecule in Sm CD27+. Panels **G** and **H** show examples of the changes in CD21 expression in Sm CD27- (TD volunteer) and the time course of the net change (percentage) of this molecule in Sm CD27-. Panels **I** and **J** show examples of the changes in CD40 expression in Um cells (TD volunteer) and the time course of the net change (median fluorescence intensity -MdFI-) of this molecule in Um cells. Histograms overlays in **E**, **G** and **I** display MdFI. The bar color indicates the net MdFI change over pre-challenge. Raw MdFI data are also shown. Panel **K** and **L** show an example of the changes in integrin α4β7 expression in Naïve cells (TD volunteer), at peak change time point after challenge and the time course of the net changes (percentage) of this molecule in Naïve cells. AroundTD is indicated by the blue rectangles with dotted lines in panels **A-D, F, H, J,** and **L**. In the same panels TD and NoTD volunteers are indicated by the blue and brown symbols, respectively. Panels **A-D, F, H, J,** and **L** display Mean ± SD.
(PDF)

S3 Fig Changes in B_M_ subsets induced by wt *S*. Typhi infection (part 2). Panels **A** and **B** show examples of the changes in CD40 expression in naive cells (TD volunteer) and the time course of the net change (MdFI) of this molecule in naive cells. Panels **C-H** show the complete time courses of various other markers evaluated in the B_M_ subsets. AroundTD is indicated by the blue rectangles with dotted lines in panels **B-H**. In the same panels TD and NoTD volunteers are indicated by the blue and brown symbols, respectively. Panels **B-H** display Mean ± SD.
(PDF)

S4 Fig Summary of changes in the expression of all molecules evaluated in the various B_M_ subsets. The statistical analyses displayed were performed using a Mixed Effects Model (as described in materials and methods). Changes (increase or decrease) in a specific marker were evaluated in TD volunteers with respect to NoTD volunteers during the specific time frames indicated. Information found in the boxes include the time frame in which the changes were identified (e.g., AroundTD and/or AfterTD); how the marker was evaluated (e.g., % net change compared to day 0) and the P value from the Mixed Effects Model analysis. Significant data are shown in highlighted boxes (light green). These boxes also include the Fig numbers in which the data is presented in the manuscript.
(PDF)

S5 Fig *S*. Typhi-LPS-specific B cells. *S*. Typhi-specific B cells were identified and concomitantly stimulated using *S*. Typhi-LPS-nanoparticles. Panel **A** displays bi-exponential plots showing the gating of LPS+ cells within the CD20+ population. Volunteers evaluated in the TD group are shown and, as expected, a high non-specific binding of LPS-nanoparticles was identified due to the “sticky” nature of LPS. Panel **B** shows the percentage of CD20+ LPS+ B cells at day 0 (pre-challenge) in TD (blue symbols) and NoTD (brown symbols) groups and demonstrate that no differences between these groups existed. Panel **C** shows the percentage of B_M_ cell subsets that are CD20+ LPS+ cells at day 0, demonstrating that no differences between TD (blue symbols) and NoTD (brown symbols) groups exist before challenge. Panels **D-F** display time courses of the frequency (cells/ml) of SmCD27+, Sm CD27- and Naïve cells. No differences between TD and NoTD groups were identified. Importantly, Um cells showed a significant difference (**Fig 6**) between TD and NoTD groups AroundTD. This is described in detail in the main body of the manuscript. In panels **D-F** AroundTD is indicated by the blue rectangles with dotted lines. In the same panels TD and NoTD volunteers are indicated by the blue and brown symbols, respectively. Panels **D-F** display Mean ± SD.
(PDF)

S6 Fig *S*. Typhi-LPS-specific B cells and their intracellular signaling profile. *S*. Typhi-specific B cells were identified and concomitantly stimulated using *S*. Typhi-LPS-nanoparticles. Changes in the signaling profile induced by typhoid were evaluated in the various B_M_ subsets. Panel **A** shows three examples of the changes in the percentage of cells phosphorylating Akt (pAkt) after challenge (day 0 vs. peak phosphorylation AroundTD) in TD volunteers. The gates were set up based on media only stimulation. The data were reported as the differential in phosphorylation between days post-challenge and day 0. Panels **B**, **C**, **D** and **E** show time courses of the net changes (percentages) in phosphorylation of signaling proteins showing significant differences between TD and NoTD volunteers AroundTD. These changes were present in SmCD27+, Sm CD27- and Naïve cells. In panels **B-E** AroundTD is indicated by the blue rectangles with dotted lines. In the same panels TD and NoTD volunteers are indicated by the blue and brown symbols, respectively. Panels **B-E** display Mean ± SD. Panel **F** shows a table summarizing the results of the signaling proteins evaluated in the different B_M_ subsets. Information found in the boxes include the time frame in which the changes were identified (e.g., TD+0h to TD+96h); how the marker was evaluated (e.g., % net change compared to day 0) and the P value from the Mixed Effects Model analysis. Significant data are shown in highlighted boxes (light green). These boxes also include the Fig numbers in which the data is presented in the manuscript.
(PDF)

## References

[1] Lozano R, Naghavi M, Foreman K, Lim S, Shibuya K, Aboyans V, et al Global and regional mortality from 235 causes of death for 20 age groups in 1990 and 2010: a systematic analysis for the Global Burden of Disease Study 2010. The Lancet. 2012;380(9859):2095–128. 10.1016/S0140-6736(12)61728-0 PMC10790329 23245604

[2] Mead PS, Slutsker L, Dietz V, McCaig LF, Bresee JS, Shapiro C, et al Food-related illness and death in the United States. Emerg Infect Dis. 1999;5(5):607–25. 10511517 10.3201/eid0505.990502 PMC2627714

[3] Mogasale V, Maskery B, Ochiai RL, Lee JS, Mogasale VV, Ramani E, et al Burden of typhoid fever in low-income and middle-income countries: a systematic, literature-based update with risk-factor adjustment. The Lancet Global health. 2014;2(10):e570–80. 10.1016/S2214-109X(14)70301-8 25304633

[4] Levine MM, Tacket CO, Sztein MB. Host-Salmonella interaction: human trials. Microbes Infect. 2001;3(14–15):1271–9. 11755415 10.1016/s1286-4579(01)01487-3

[5] Pasetti MF, Levine MM, Sztein MB. Animal models paving the way for clinical trials of attenuated Salmonella enterica serovar Typhi live oral vaccines and live vectors. Vaccine. 2003;21(5–6):401–18. 12531639 10.1016/s0264-410x(02)00472-3

[6] Hornick RB, Greisman SE, Woodward TE, DuPont HL, Dawkins AT, Snyder MJ. Typhoid Fever: Pathogenesis and Immunologic Control. New England Journal of Medicine. 1970;283(13):686–91. 4916913 10.1056/NEJM197009242831306

[7] Gilman RH, Hornick RB, Woodward WE, DuPont HL, Snyder MJ, Levine MM, et al Evaluation of a UDP-Glucose-4-Epimeraseless Mutant of Salmonella typhi as a Live Oral Vaccine. Journal of Infectious Diseases. 1977;136(6):717–23. 925379 10.1093/infdis/136.6.717

[8] Hornick RB, Woodward TE. Appraisal of typhoid vaccine in experimentally infected human subjects. Transactions of the American Clinical and Climatological Association. 1967;78:70–8. 6028241 PMC2441150

[9] Gilman RH, Hornick RB. Duodenal isolation of Salmonella typhi by string capsule in acute typhoid fever. J Clin Microbiol. 1976;3(4):456–7. 770502 10.1128/jcm.3.4.456-457.1976 PMC274324

[10] Waddington CS, Darton TC, Woodward WE, Angus B, Levine MM, Pollard AJ. Advancing the management and control of typhoid fever: A review of the historical role of human challenge studies. Journal of Infection. 2014;68(5):405–18. 10.1016/j.jinf.2014.01.006 24491597

[11] Waddington CS, Darton TC, Jones C, Haworth K, Peters A, John T, et al An Outpatient, Ambulant-Design, Controlled Human Infection Model Using Escalating Doses of Salmonella Typhi Challenge Delivered in Sodium Bicarbonate Solution. Clinical Infectious Diseases. 2014;58(9):1230–40. 10.1093/cid/ciu078 24519873 PMC3982839

[12] Sanz I, Wei C, Lee FE, Anolik J. Phenotypic and functional heterogeneity of human memory B cells. Semin Immunol. 2008;20(1):67–82. 10.1016/j.smim.2007.12.006 18258454 PMC2440717

[13] Wei C, Anolik J, Cappione A, Zheng B, Pugh-Bernard A, Brooks J, et al A New Population of Cells Lacking Expression of CD27 Represents a Notable Component of the B Cell Memory Compartment in Systemic Lupus Erythematosus. The Journal of Immunology. 2007;178(10):6624–33. 17475894 10.4049/jimmunol.178.10.6624

[14] Qian Y, Wei C, Eun-Hyung Lee F, Campbell J, Halliley J, Lee JA, et al Elucidation of seventeen human peripheral blood B-cell subsets and quantification of the tetanus response using a density-based method for the automated identification of cell populations in multidimensional flow cytometry data. Cytometry B Clin Cytom. 2010;78 Suppl 1:S69–82. 10.1002/cyto.b.20554 20839340 PMC3084630

[15] Blair PA, Noreña LY, Flores-Borja F, Rawlings DJ, Isenberg DA, Ehrenstein MR, et al CD19+CD24hiCD38hi B Cells Exhibit Regulatory Capacity in Healthy Individuals but Are Functionally Impaired in Systemic Lupus Erythematosus Patients. Immunity. 2010;32(1):129–40. 10.1016/j.immuni.2009.11.009 20079667

[16] Mauri C, Bosma A. Immune Regulatory Function of B Cells. Annu Rev Immunol. 2012;30(1):221–41. 22224776 10.1146/annurev-immunol-020711-074934

[17] Narvaez CF, Feng N, Vasquez C, Sen A, Angel J, Greenberg HB, et al Human rotavirus-specific IgM Memory B cells have differential cloning efficiencies and switch capacities and play a role in antiviral immunity in vivo. J Virol. 2012;86(19):10829–40. 10.1128/JVI.01466-12 22855480 PMC3457288

[18] Khaskhely N, Mosakowski J, Thompson RS, Khuder S, Smithson SL, Westerink MAJ. Phenotypic Analysis of Pneumococcal Polysaccharide-Specific B Cells. The Journal of Immunology. 2012;188(5):2455–63. 10.4049/jimmunol.1102809 22271652 PMC3288481

[19] Taylor JJ, Jenkins MK, Pape KA. Heterogeneity in the differentiation and function of memory B cells. Trends in immunology. 2012;33(12):590–7. 10.1016/j.it.2012.07.005 22920843 PMC3505266

[20] Kruetzmann S, Rosado MM, Weber H, Germing U, Tournilhac O, Peter H-H, et al Human Immunoglobulin M Memory B Cells Controlling Streptococcus pneumoniae Infections Are Generated in the Spleen. The Journal of Experimental Medicine. 2003;197(7):939–45. 12682112 10.1084/jem.20022020 PMC2193885

[21] So NSY, Ostrowski MA, Gray-Owen SD. Vigorous Response of Human Innate Functioning IgM Memory B Cells upon Infection by Neisseria gonorrhoeae. The Journal of Immunology. 2012;188(8):4008–22. 10.4049/jimmunol.1100718 22427638

[22] Levine MM, Galen J. E., Tacket E. M., Barry M. F., Pasetti M. F., and Sztein M. B. Attenuated strains of Salmonella enterica serovar Typhi as Live Oral Fever In: Levine MM, Kaper J. B., Lui M. and Good M., editor. New Generation Vaccines. New York: Marcel Dekker; 2004 p. 479.

[23] de Wit J, Souwer Y, Jorritsma T, Klaasse Bos H, ten Brinke A, Neefjes J, et al Antigen-Specific B Cells Reactivate an Effective Cytotoxic T Cell Response against Phagocytosed *Salmonella* through Cross-Presentation. PLoS ONE. 2010;5(9):e13016 10.1371/journal.pone.0013016 20885961 PMC2946406

[24] Mastroeni P, Simmons C, Fowler R, Hormaeche CE, Dougan G. Igh-6(-/-) (B-cell-deficient) mice fail to mount solid acquired resistance to oral challenge with virulent Salmonella enterica serovar typhimurium and show impaired Th1 T-cell responses to Salmonella antigens. Infect Immun. 2000;68(1):46–53. 10603367 10.1128/iai.68.1.46-53.2000 PMC97100

[25] Mittrucker HW, Raupach B, Kohler A, Kaufmann SH. Cutting edge: role of B lymphocytes in protective immunity against Salmonella typhimurium infection. J Immunol. 2000;164(4):1648–52. 10657605 10.4049/jimmunol.164.4.1648

[26] Lopez-Medina M, Perez-Lopez A, Alpuche-Aranda C, Ortiz-Navarrete V. Salmonella modulates B cell biology to evade CD8(+) T cell-mediated immune responses. Front Immunol. 2014;5:586 10.3389/fimmu.2014.00586 25484884 PMC4240163

[27] McSorley SJ, Jenkins MK. Antibody is required for protection against virulent but not attenuated Salmonella enterica serovar typhimurium. Infect Immun. 2000;68(6):3344–8. 10816483 10.1128/iai.68.6.3344-3348.2000 PMC97596

[28] Barr TA, Brown S, Mastroeni P, Gray D. B cell intrinsic MyD88 signals drive IFN-gamma production from T cells and control switching to IgG2c. J Immunol. 2009;183(2):1005–12. 10.4049/jimmunol.0803706 19542370 PMC7224988

[29] Barr TA, Brown S, Mastroeni P, Gray D. TLR and B cell receptor signals to B cells differentially program primary and memory Th1 responses to Salmonella enterica. J Immunol. 2010;185(5):2783–9. 10.4049/jimmunol.1001431 20675594 PMC3745605

[30] Ruprecht CR, Lanzavecchia A. Toll-like receptor stimulation as a third signal required for activation of human naive B cells. Eur J Immunol. 2006;36(4):810–6. 16541472 10.1002/eji.200535744

[31] Nothelfer K, Sansonetti PJ, Phalipon A. Pathogen manipulation of B cells: the best defence is a good offence. Nature reviews Microbiology. 2015;13(3):173–84. 10.1038/nrmicro3415 25659322

[32] Souwer Y, Griekspoor A, Jorritsma T, de Wit J, Janssen H, Neefjes J, et al B cell receptor-mediated internalization of salmonella: a novel pathway for autonomous B cell activation and antibody production. J Immunol. 2009;182(12):7473–81. 10.4049/jimmunol.0802831 19494270

[33] Souwer Y, Griekspoor A, de Wit J, Martinoli C, Zagato E, Janssen H, et al Selective infection of antigen-specific B lymphocytes by Salmonella mediates bacterial survival and systemic spreading of infection. PLoS One. 2012;7(11):e50667 10.1371/journal.pone.0050667 23209805 PMC3510171

[34] Rosales-Reyes R, Perez-Lopez A, Sanchez-Gomez C, Hernandez-Mote RR, Castro-Eguiluz D, Ortiz-Navarrete V, et al Salmonella infects B cells by macropinocytosis and formation of spacious phagosomes but does not induce pyroptosis in favor of its survival. Microb Pathog. 2012;52(6):367–74. 10.1016/j.micpath.2012.03.007 22475626

[35] Neves P, Lampropoulou V, Calderon-Gomez E, Roch T, Stervbo U, Shen P, et al Signaling via the MyD88 adaptor protein in B cells suppresses protective immunity during Salmonella typhimurium infection. Immunity. 2010;33(5):777–90. 10.1016/j.immuni.2010.10.016 21093317

[36] Shen P, Roch T, Lampropoulou V, O'Connor RA, Stervbo U, Hilgenberg E, et al IL-35-producing B cells are critical regulators of immunity during autoimmune and infectious diseases. Nature. 2014;507(7492):366–70. 10.1038/nature12979 24572363 PMC4260166

[37] Perez-Lopez A, Rosales-Reyes R, Alpuche-Aranda CM, Ortiz-Navarrete V. Salmonella downregulates Nod-like receptor family CARD domain containing protein 4 expression to promote its survival in B cells by preventing inflammasome activation and cell death. J Immunol. 2013;190(3):1201–9. 10.4049/jimmunol.1200415 23284055

[38] Wahid R, Pasetti MF, Maciel M Jr., Simon JK, Tacket CO, Levine MM, et al Oral priming with Salmonella Typhi vaccine strain CVD 909 followed by parenteral boost with the S. Typhi Vi capsular polysaccharide vaccine induces CD27+IgD-S. Typhi-specific IgA and IgG B memory cells in humans. ClinImmunol. 2011;138(2):187–200. 10.1016/j.clim.2010.11.006 PMC3035995 21146460

[39] Wahid R, Simon R, Zafar SJ, Levine MM, Sztein MB. Live oral typhoid vaccine Ty21a induces cross-reactive humoral immune responses against Salmonella enterica serovar Paratyphi A and S. Paratyphi B in humans. Clin Vaccine Immunol. 2012;19(6):825–34. 10.1128/CVI.00058-12 22492745 PMC3370435

[40] Toapanta FR, Bernal PJ, Fresnay S, Darton TC, Jones C, Waddington CS, et al Oral Wild-Type Salmonella Typhi Challenge Induces Activation of Circulating Monocytes and Dendritic Cells in Individuals Who Develop Typhoid Disease. PLoS neglected tropical diseases. 2015;9(6):e0003837 10.1371/journal.pntd.0003837 26065687 PMC4465829

[41] McArthur MA, Sztein MB. Heterogeneity of Multifunctional IL-17A Producing *S*. Typhi-Specific CD8+ T Cells in Volunteers following Ty21a Typhoid Immunization. PLoS ONE. 2012;7(6):e38408 10.1371/journal.pone.0038408 22679502 PMC3367967

[42] Toapanta FR, Ross TM. Impaired immune responses in the lungs of aged mice following influenza infection. Respir Res. 2009;10:112 10.1186/1465-9921-10-112 19922665 PMC2785782

[43] Toapanta FR, Bernal PJ, Sztein MB. Diverse phosphorylation patterns of B cell receptor-associated signaling in naive and memory human B cells revealed by phosphoflow, a powerful technique to study signaling at the single cell level. Front Cell Infect Microbiol. 2012;2:128 10.3389/fcimb.2012.00128 23087912 PMC3473368

[44] Betanzos CM, Gonzalez-Moa M, Johnston SA, Svarovsky SA. Facile labeling of lipoglycans with quantum dots. Biochemical and Biophysical Research Communications. 2009;380(1):1–4. 10.1016/j.bbrc.2008.12.167 19150336

[45] Anderson RE, Chan WCW. Systematic Investigation of Preparing Biocompatible, Single, and Small ZnS-Capped CdSe Quantum Dots with Amphiphilic Polymers. ACS Nano. 2008;2(7):1341–52. 10.1021/nn700450g 19206301

[46] Dubertret B, Skourides P, Norris DJ, Noireaux V, Brivanlou AH, Libchaber A. In Vivo Imaging of Quantum Dots Encapsulated in Phospholipid Micelles. Science. 2002;298(5599):1759–62. 12459582 10.1126/science.1077194

[47] Grammer AC, Lipsky PE. CD154-CD40 interactions mediate differentiation to plasma cells in healthy individuals and persons with systemic lupus erythematosus. Arthritis Rheum. 2002;46(6):1417–29. 12115170 10.1002/art.10287

[48] Iwata M, Hirakiyama A, Eshima Y, Kagechika H, Kato C, Song SY. Retinoic acid imprints gut-homing specificity on T cells. Immunity. 2004;21(4):527–38. 15485630 10.1016/j.immuni.2004.08.011

[49] Mora JR, Iwata M, Eksteen B, Song SY, Junt T, Senman B, et al Generation of gut-homing IgA-secreting B cells by intestinal dendritic cells. Science. 2006;314(5802):1157–60. 17110582 10.1126/science.1132742

[50] Johansson-Lindbom B, Svensson M, Pabst O, Palmqvist C, Marquez G, Forster R, et al Functional specialization of gut CD103+ dendritic cells in the regulation of tissue-selective T cell homing. J Exp Med. 2005;202(8):1063–73. 16216890 10.1084/jem.20051100 PMC2213212

[51] Hammerschmidt SI, Ahrendt M, Bode U, Wahl B, Kremmer E, Forster R, et al Stromal mesenteric lymph node cells are essential for the generation of gut-homing T cells in vivo. J Exp Med. 2008;205(11):2483–90. 10.1084/jem.20080039 18852290 PMC2571923

[52] Toapanta FR, Simon JK, Barry EM, Pasetti MF, Levine MM, Kotloff KL, et al Gut-homing conventional plasmablasts and CD27- plasmablasts elicited after a short time exposure to an oral live attenuated Shigella vaccine candidate in humans. Frontiers in Immunology. 2014;5. 10.3389/fimmu.2014.00374 PMC4138503 25191323

[53] Weller S, Braun MC, Tan BK, Rosenwald A, Cordier C, Conley ME, et al Human blood IgM “memory” B cells are circulating splenic marginal zone B cells harboring a prediversified immunoglobulin repertoire. Blood. 2004;104(12):3647–54. 15191950 10.1182/blood-2004-01-0346 PMC2590648

[54] Shi Y, Agematsu K, Ochs HD, Sugane K. Functional analysis of human memory B-cell subpopulations: IgD+CD27+ B cells are crucial in secondary immune response by producing high affinity IgM. Clin Immunol. 2003;108(2):128–37. 12921759 10.1016/s1521-6616(03)00092-5

[55] Isnardi I, Ng YS, Menard L, Meyers G, Saadoun D, Srdanovic I, et al Complement receptor 2/CD21- human naive B cells contain mostly autoreactive unresponsive clones. Blood. 2010;115(24):5026–36. 10.1182/blood-2009-09-243071 20231422 PMC3373152

[56] Saadoun D, Terrier B, Bannock J, Vazquez T, Massad C, Kang I, et al Expansion of autoreactive unresponsive CD21-/low B cells in Sjogren's syndrome-associated lymphoproliferation. Arthritis Rheum. 2013;65(4):1085–96. 10.1002/art.37828 23279883 PMC4479193

[57] Jacobson KH, Gunsolus IL, Kuech TR, Troiano JM, Melby ES, Lohse SE, et al Lipopolysaccharide Density and Structure Govern the Extent and Distance of Nanoparticle Interaction with Actual and Model Bacterial Outer Membranes. Environmental science & technology. 2015;49(17):10642–50. 26207769 10.1021/acs.est.5b01841 PMC4643684

