# Supplementary figures and images for: Oral Challenge with Wild-Type Salmonella Typhi Induces Distinct Changes in B Cell Subsets in Individuals Who Develop Typhoid Disease
Source: PLoS Negl Trop Dis. 2016 Jun 14;10(6):e0004766. doi: 10.1371/journal.pntd.0004766 (PMC4907489; doi:10.1371/journal.pntd.0004766)

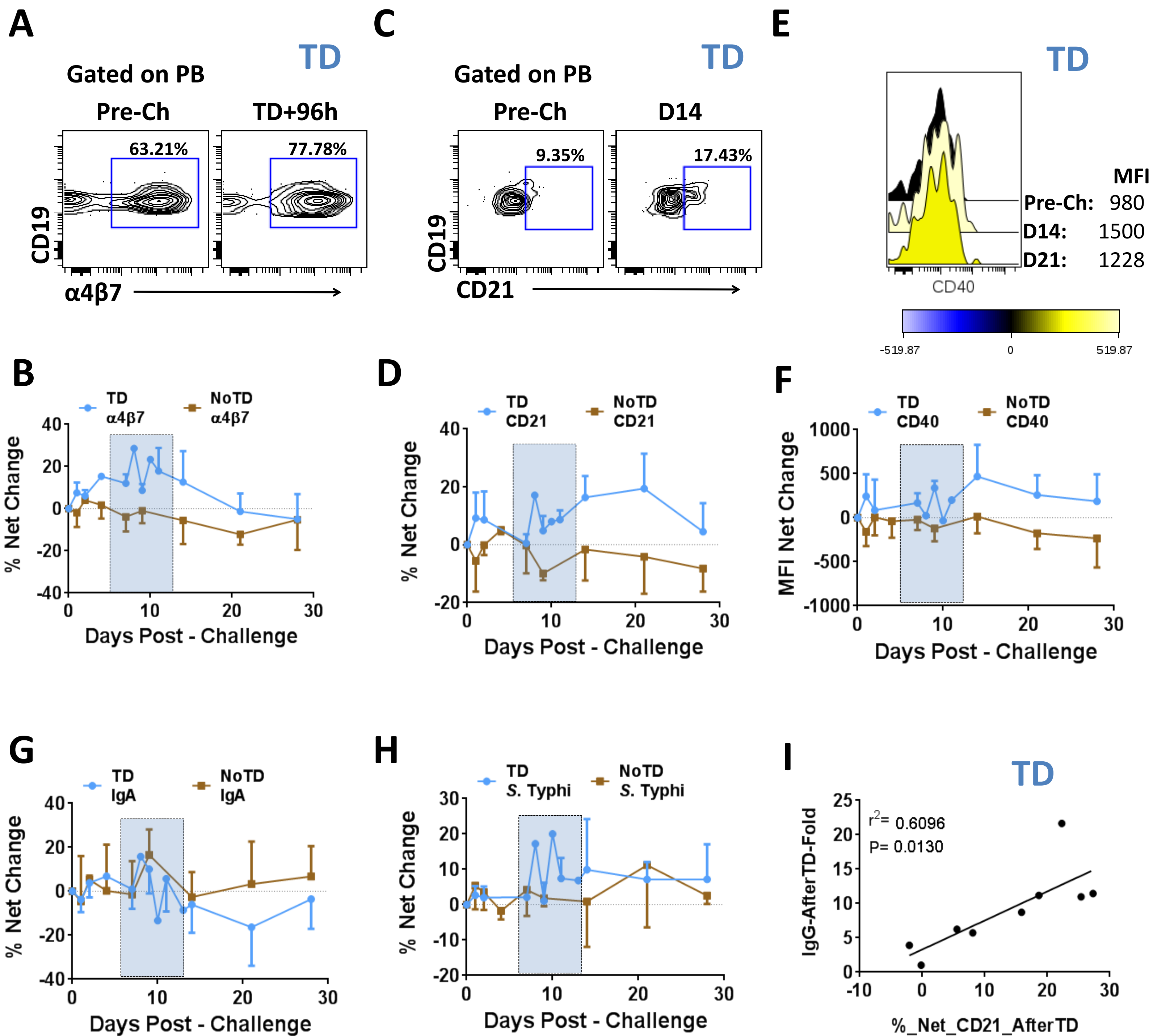

Supplement: S1 Fig — Panels A and B show examples of the changes in the expression of integrin α4β7 and the time course of the net change (percentage) of this molecule in PBs. TD and NoTD volunteers are indicated by the blue and brown symbols, respectively. Panels C and D show examples of the changes in the expression of CD21 and the time course of the net change (percentage) of this molecule in PBs. Panels E and F show examples of the changes in the expression of CD40 and the time course of the net change (MFI) of this molecule in PBs. Graphs A, C and E show the data before challenge and peak increases after-challenge. In Panel E the bar color bar indicates the net MFI change over pre-challenge. Also sown are the raw MFI values. Panels G and H show the complete time courses of IgA expression and S. Typhi binding in PB in TD (blue symbols) and NoTD (brown symbols) volunteers. AroundTD is indicated by the blue rectangles with dotted lines in panels B, D, F, G, and H. Panels B, D, F, G, and H display Mean ± SD. Panel I displays the Spearman correlation results between PB CD21+ cells (% net change over day 0; AfterTD time frame) and the anti-flagellin IgG antibody titer (fold-increase over day 0; After TD time frame) in TD volunteers. The AfterTD time frame for the antibody titers included data from days 14, 28 and 60. (PDF) [file pntd.0004766.s001.pdf]

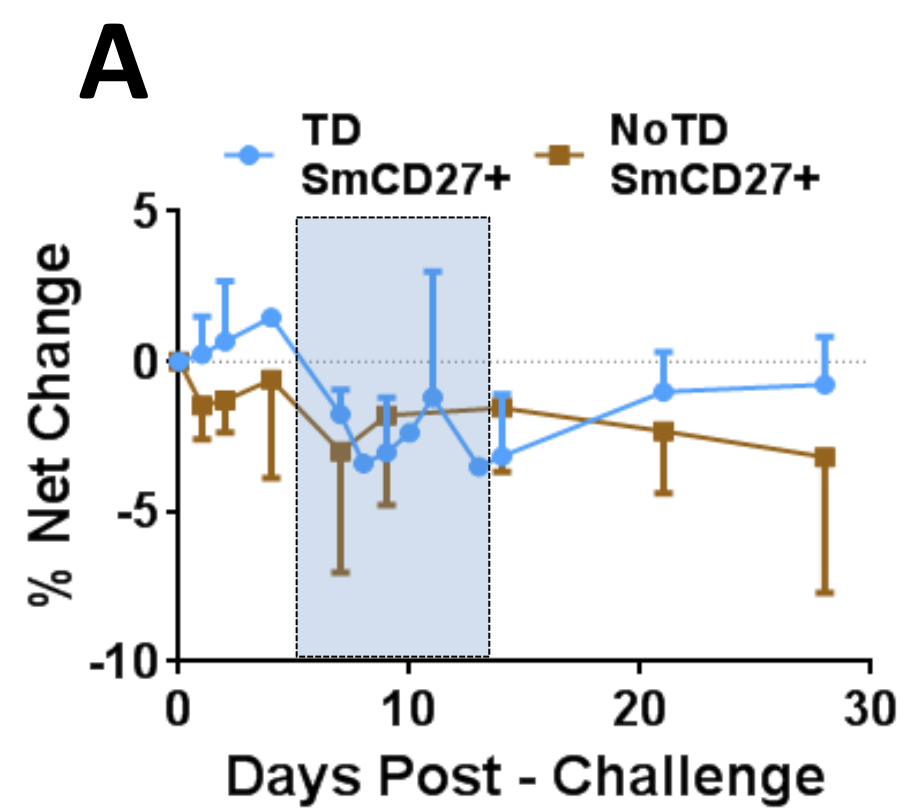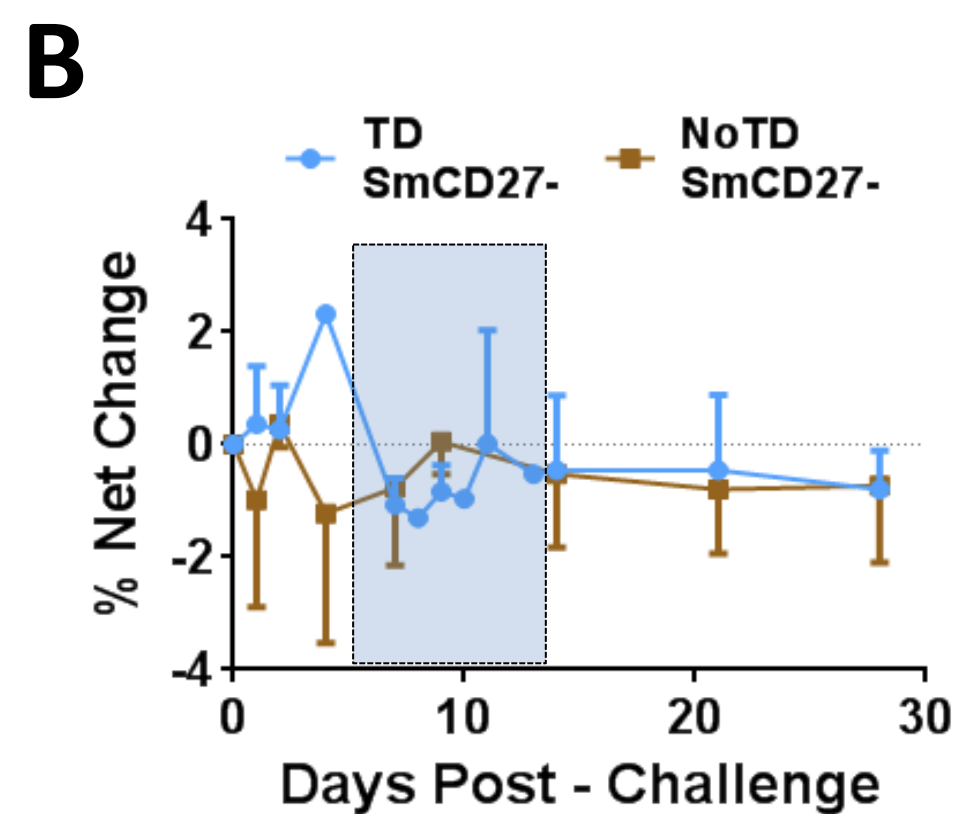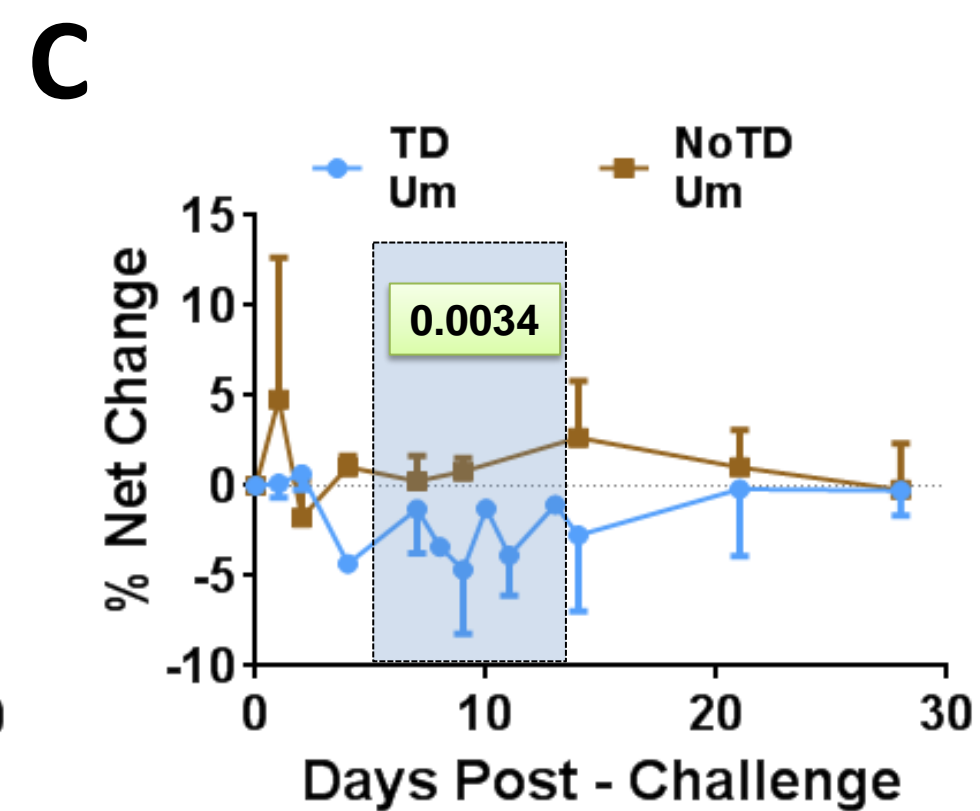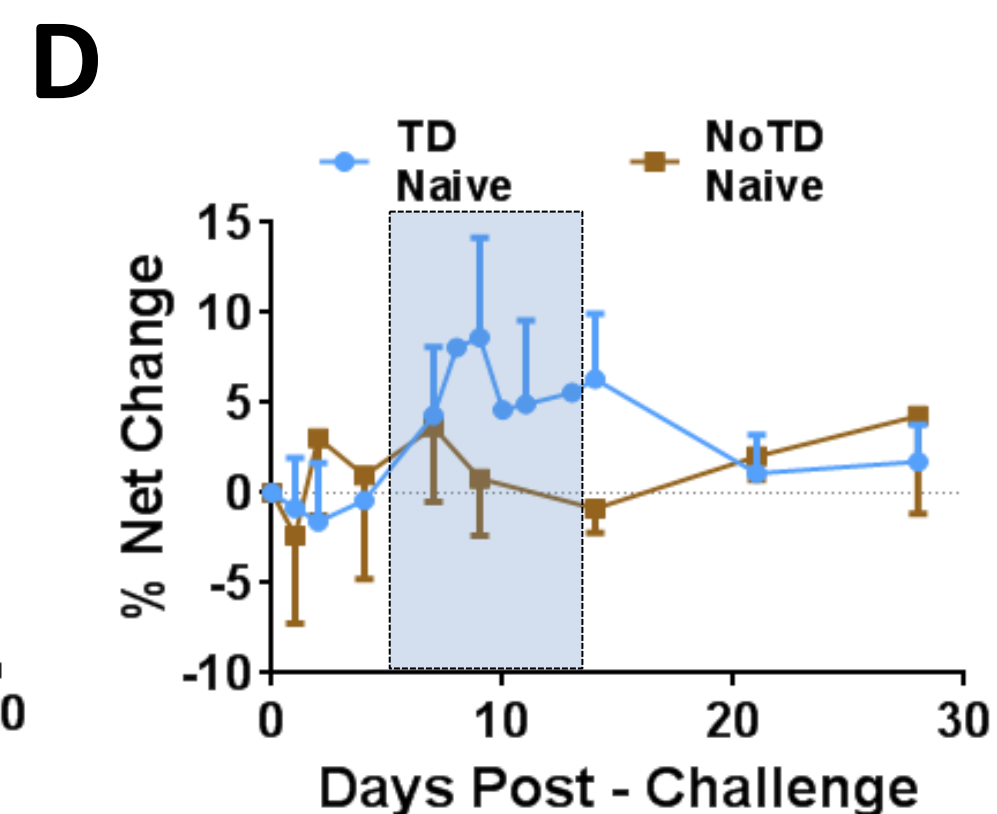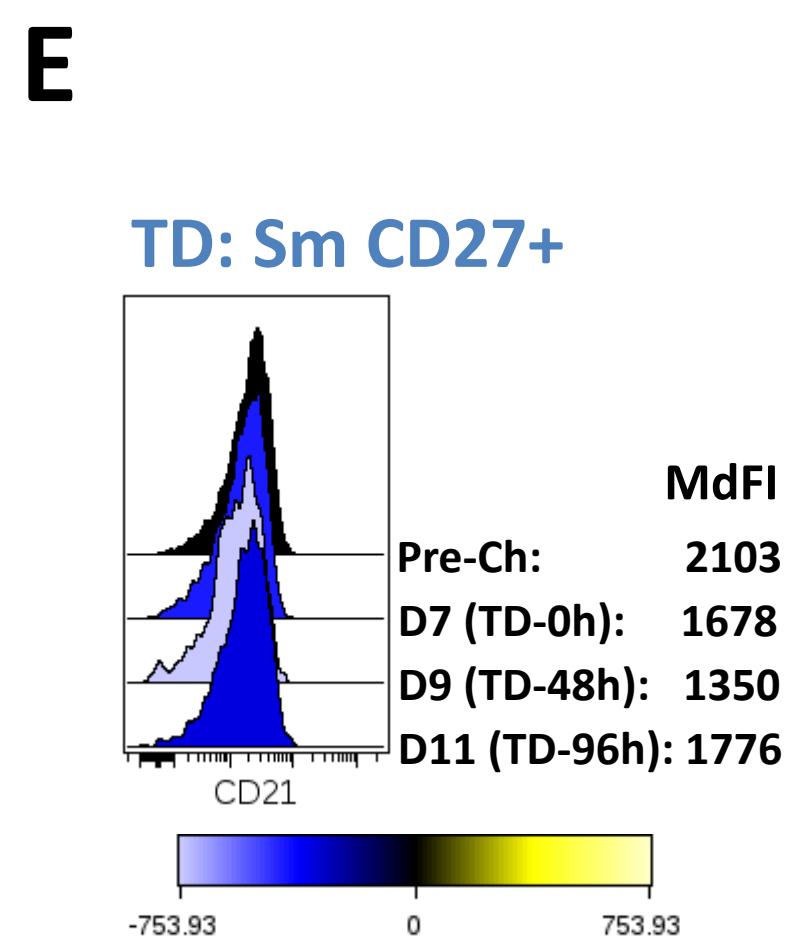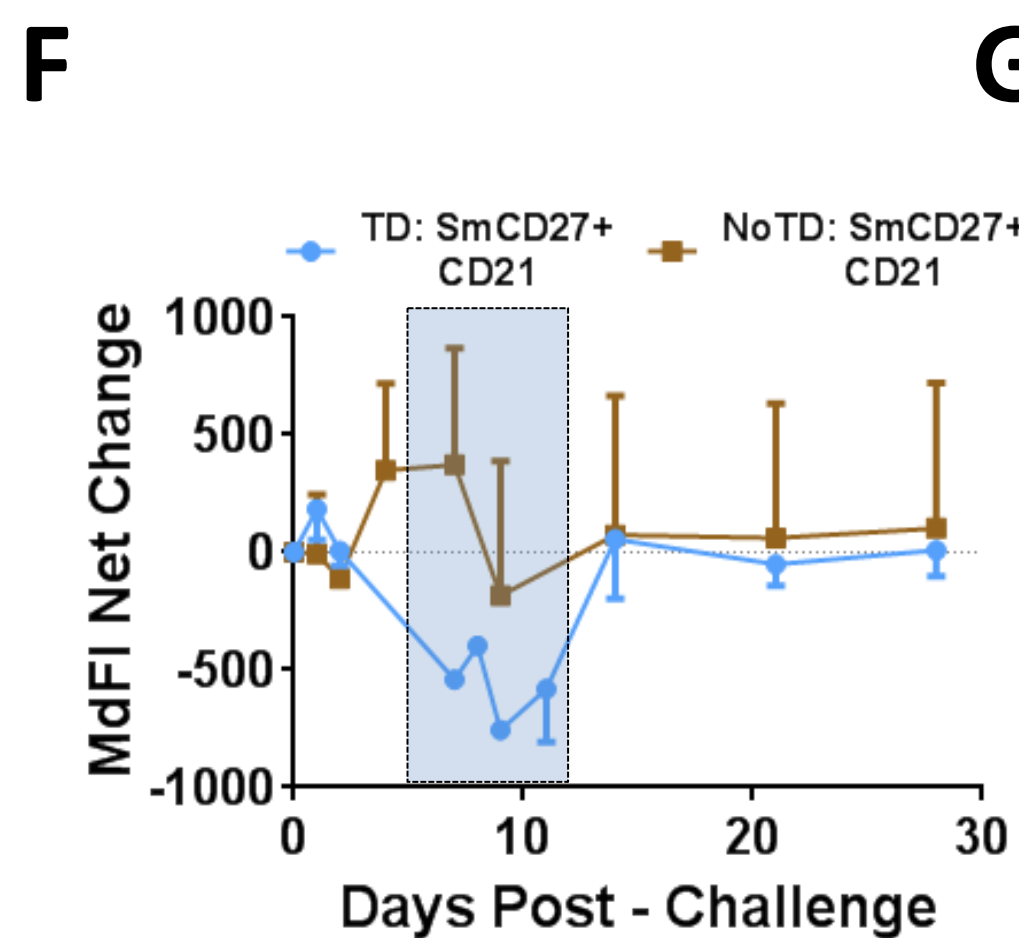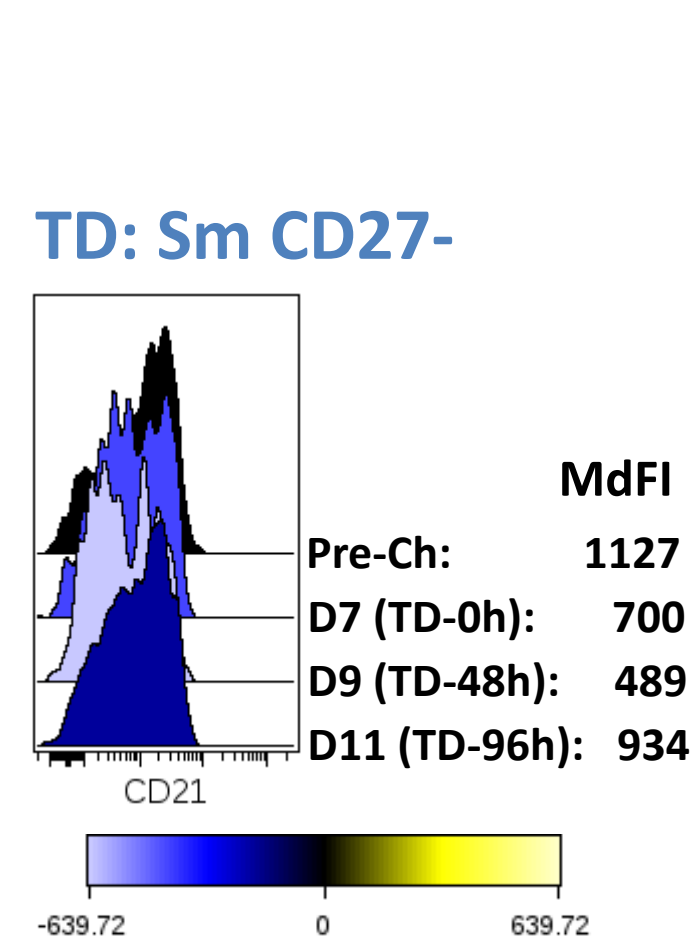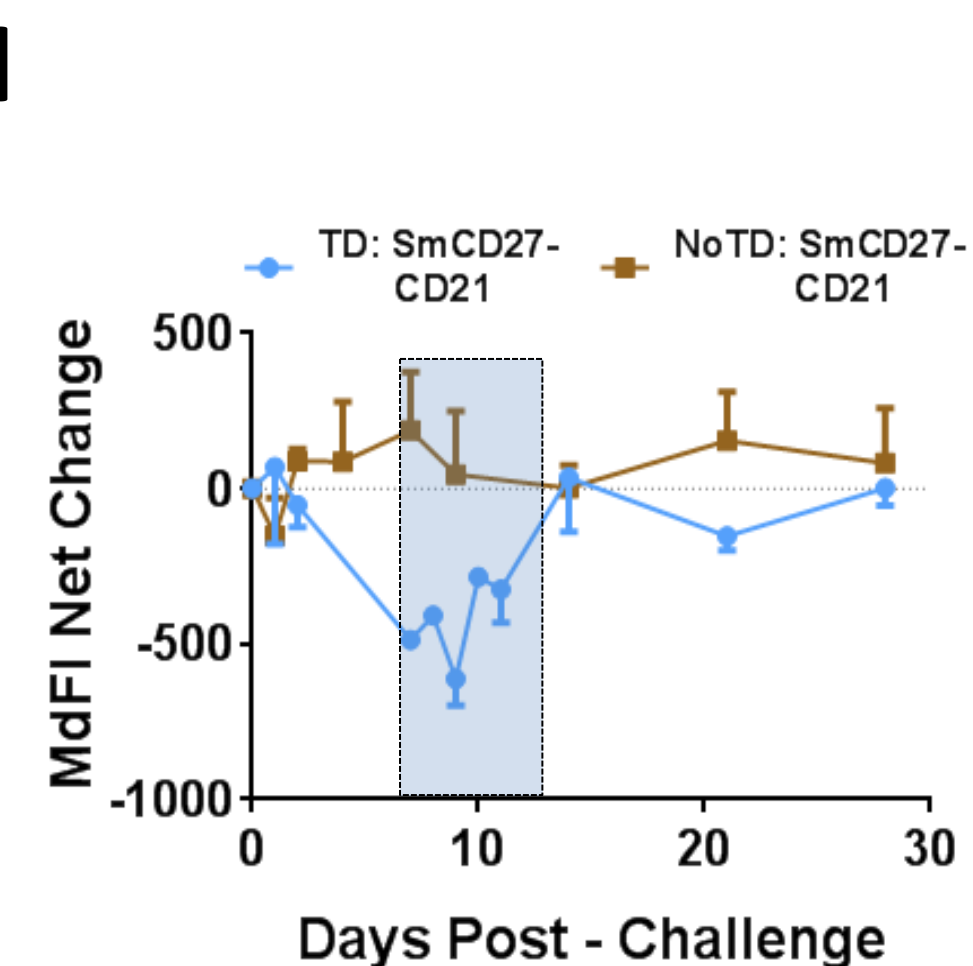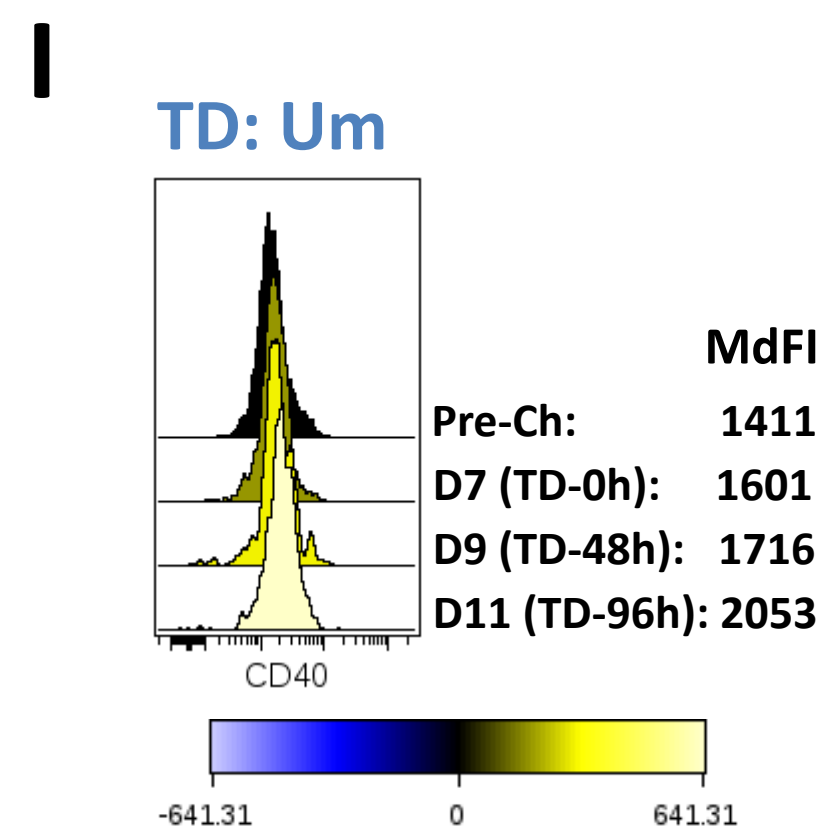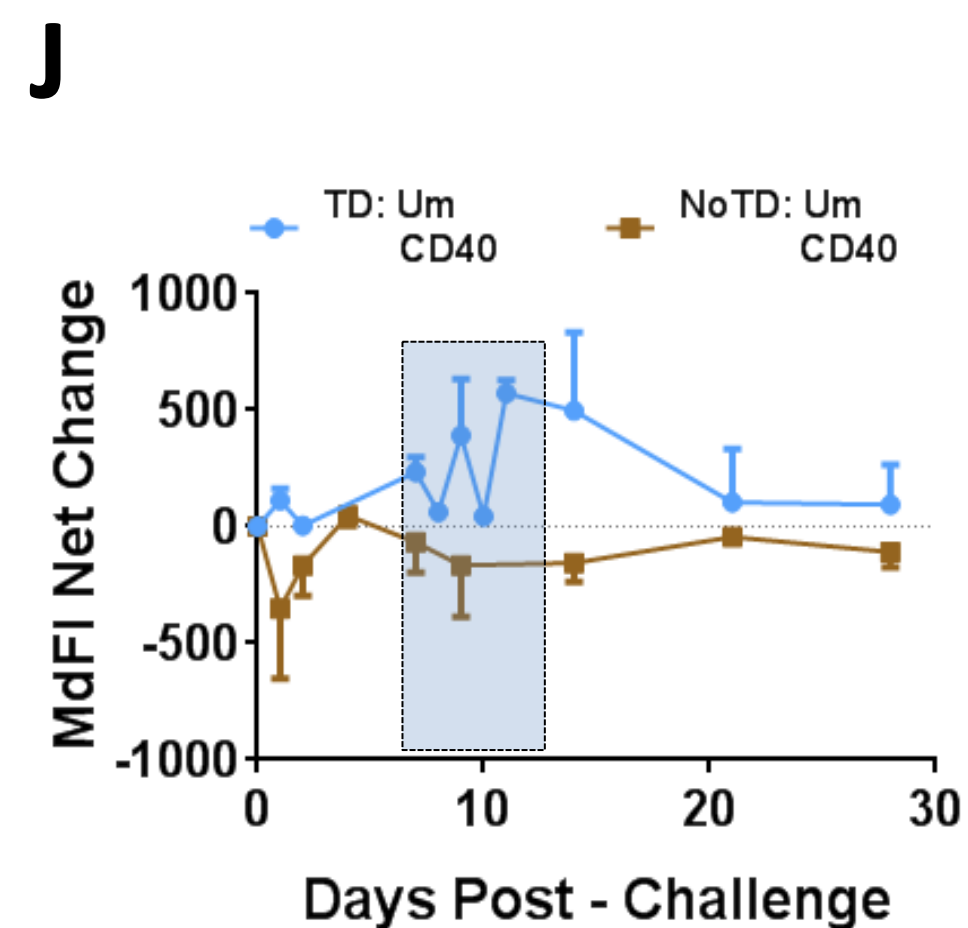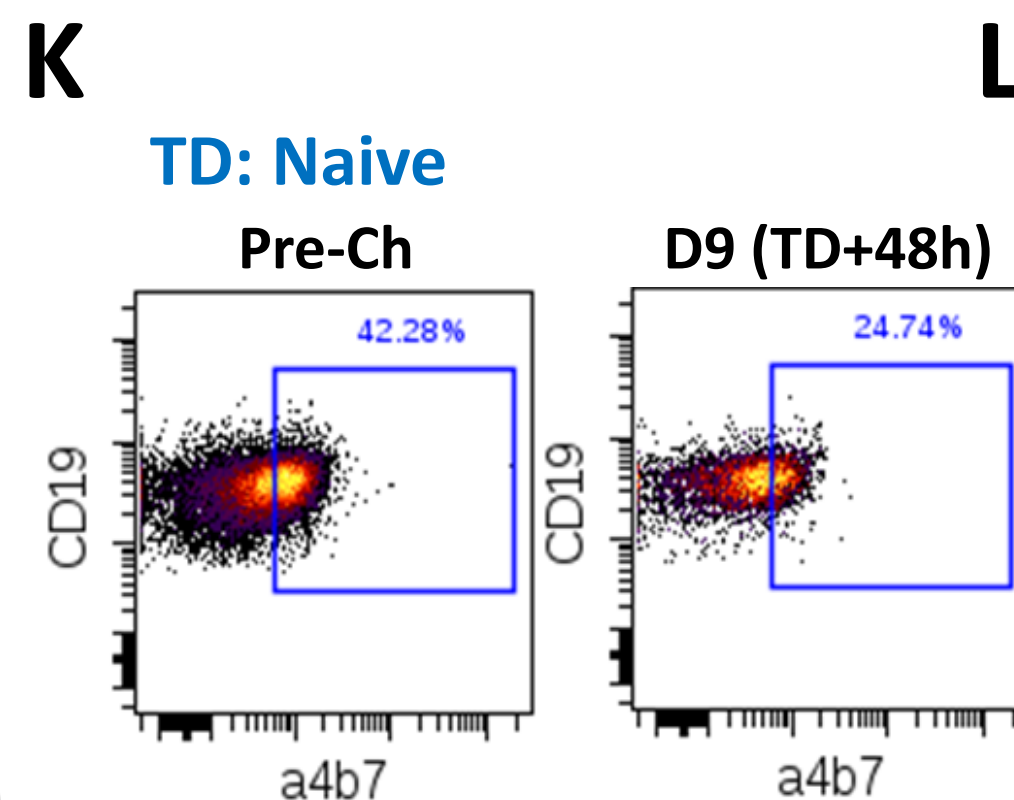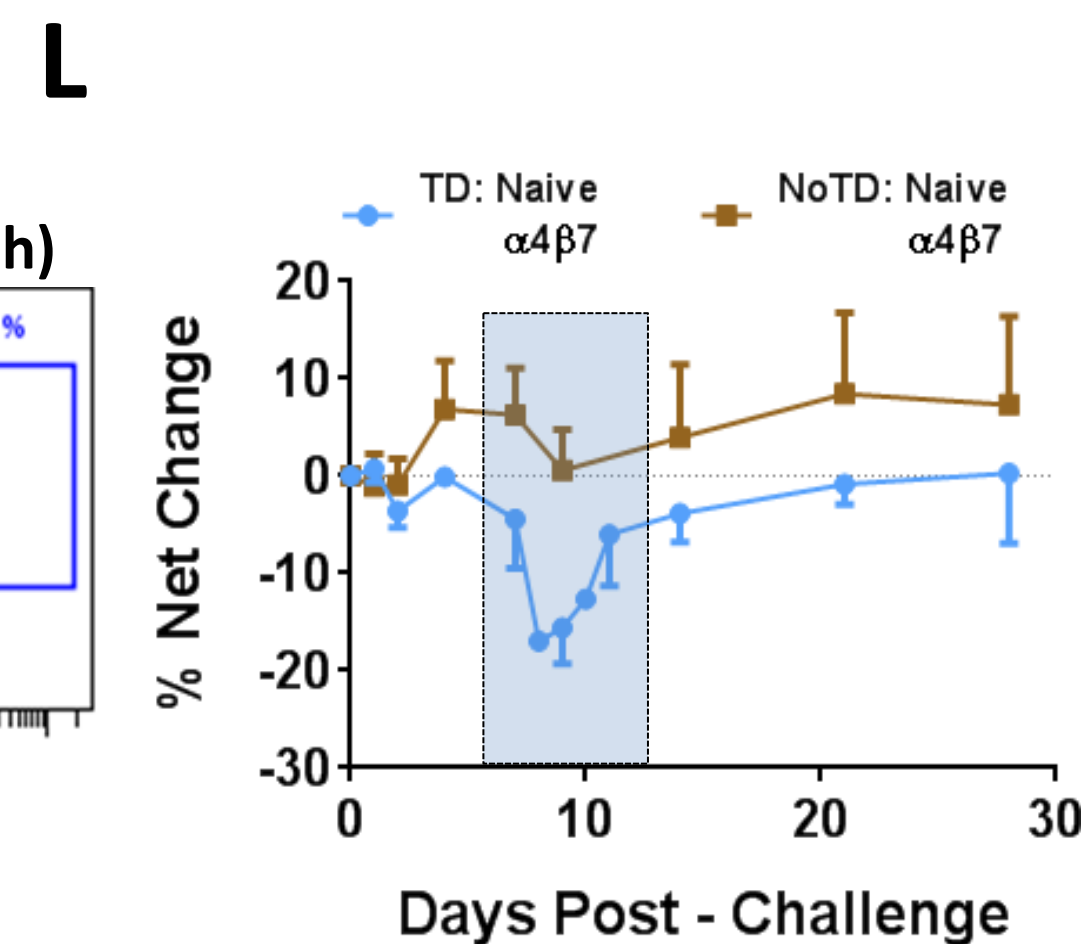

Supplement: S2 Fig — Panels A-D show the time course of the net changes (percentage) of the various BM populations. The p value (mixed effects model) for significant differences between TD and NoTD volunteers at AroundTD is indicated in the green rectangle. Panels E and F show examples of the changes in CD21 expression in Sm CD27+ (TD volunteer) and the time course of the net change (percentage) of this molecule in Sm CD27+. Panels G and H show examples of the changes in CD21 expression in Sm CD27- (TD volunteer) and the time course of the net change (percentage) of this molecule in Sm CD27-. Panels I and J show examples of the changes in CD40 expression in Um cells (TD volunteer) and the time course of the net change (median fluorescence intensity -MdFI-) of this molecule in Um cells. Histograms overlays in E, G and I display MdFI. The bar color indicates the net MdFI change over pre-challenge. Raw MdFI data are also shown. Panel K and L show an example of the changes in integrin α4β7 expression in Naïve cells (TD volunteer), at peak change time point after challenge and the time course of the net changes (percentage) of this molecule in Naïve cells. AroundTD is indicated by the blue rectangles with dotted lines in panels A-D, F, H, J, and L. In the same panels TD and NoTD volunteers are indicated by the blue and brown symbols, respectively. Panels A-D, F, H, J, and L display Mean ± SD. (PDF) [file pntd.0004766.s002.pdf]

**A**

TD: Naive

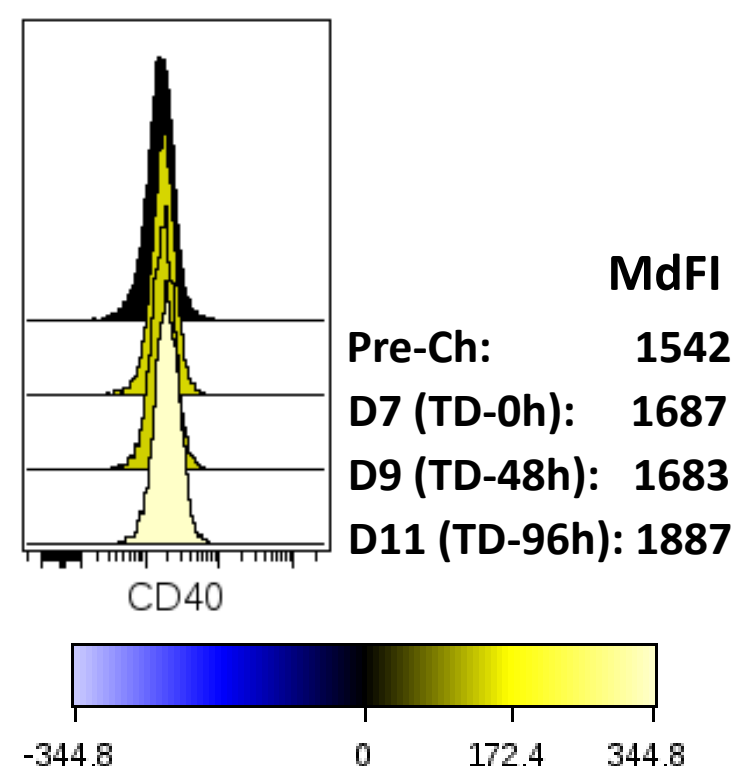**B**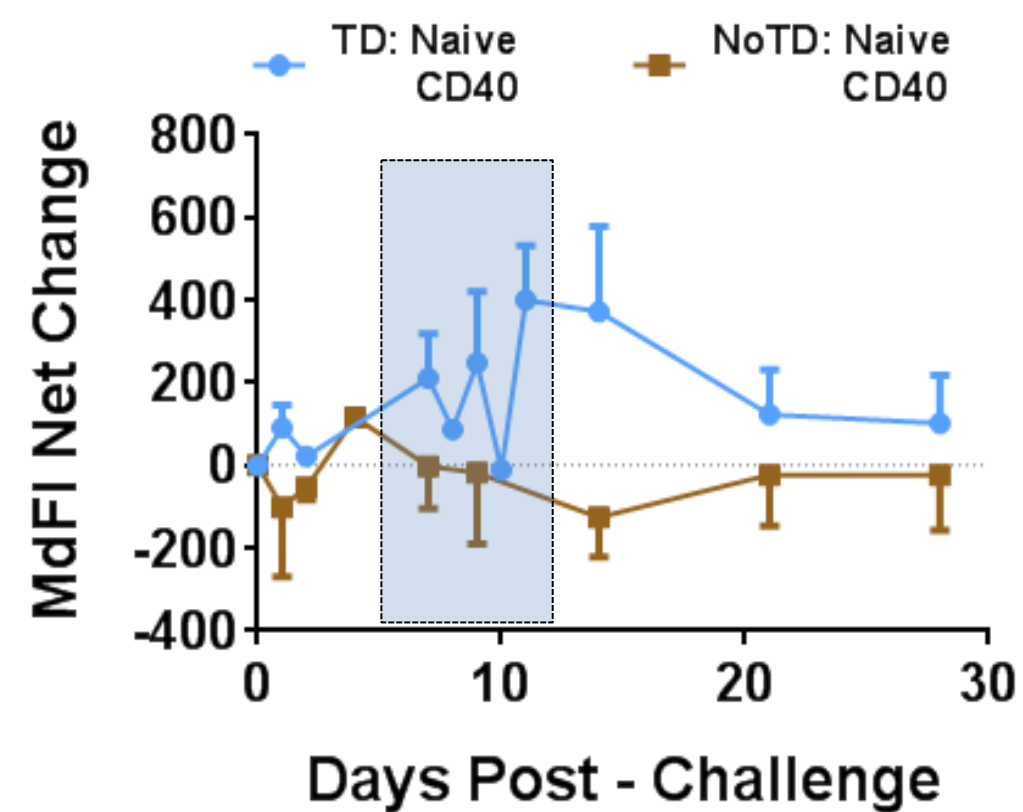**C**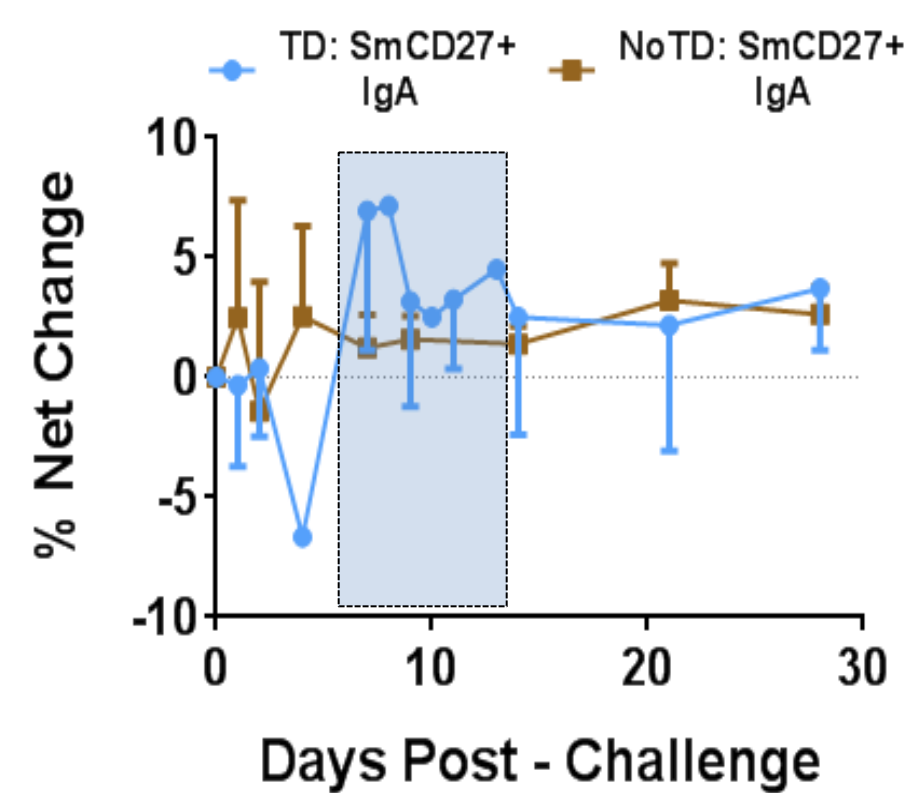**D**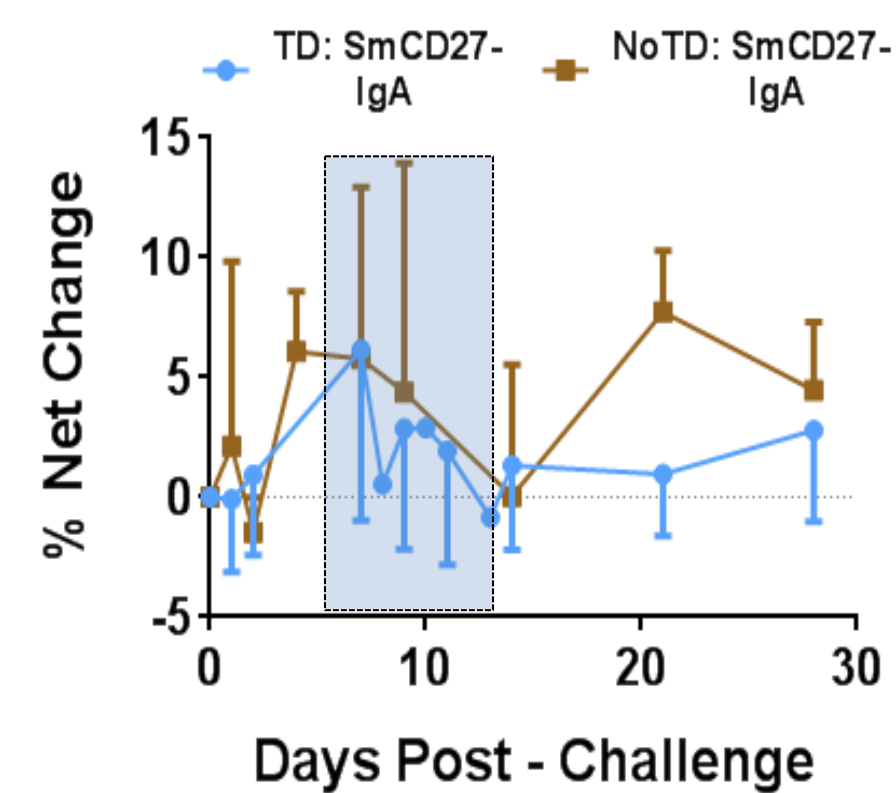**E**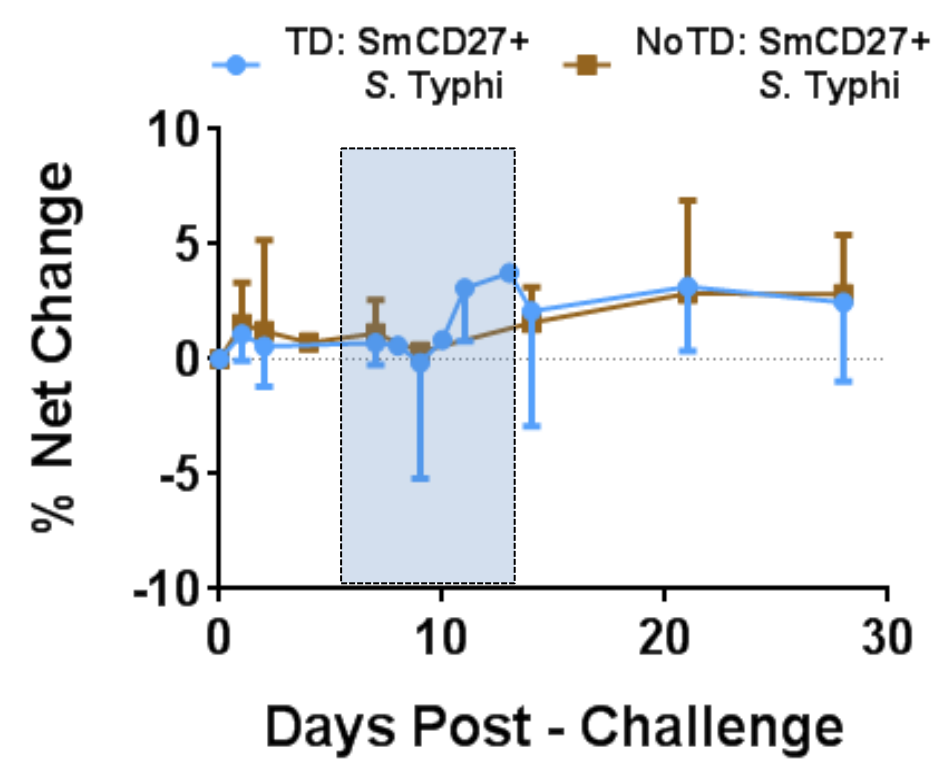**F**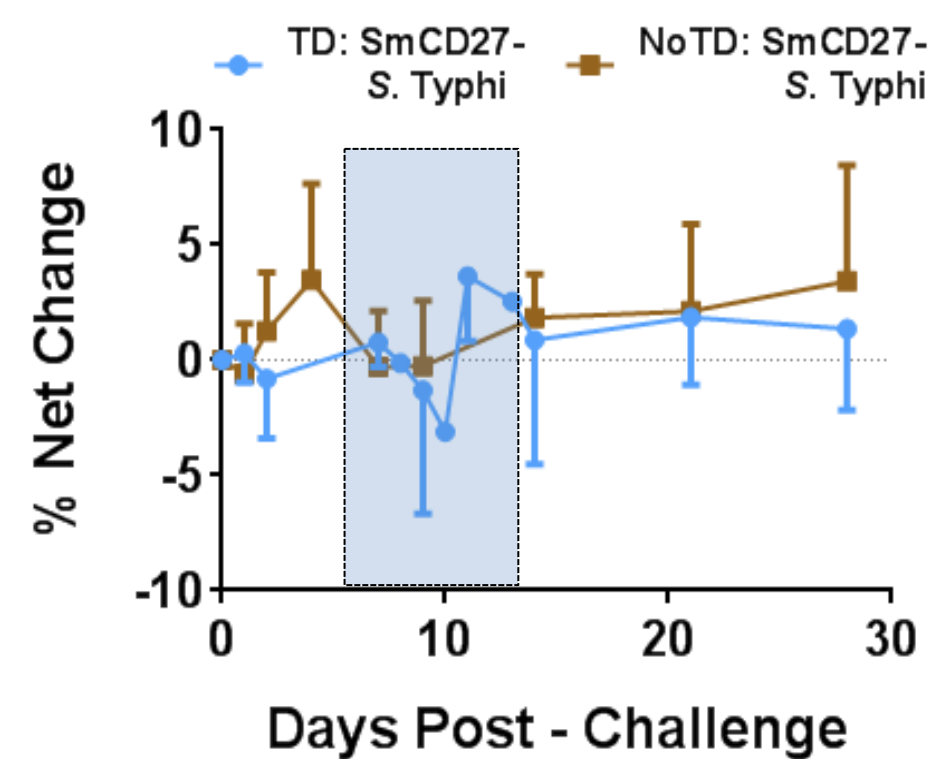**G**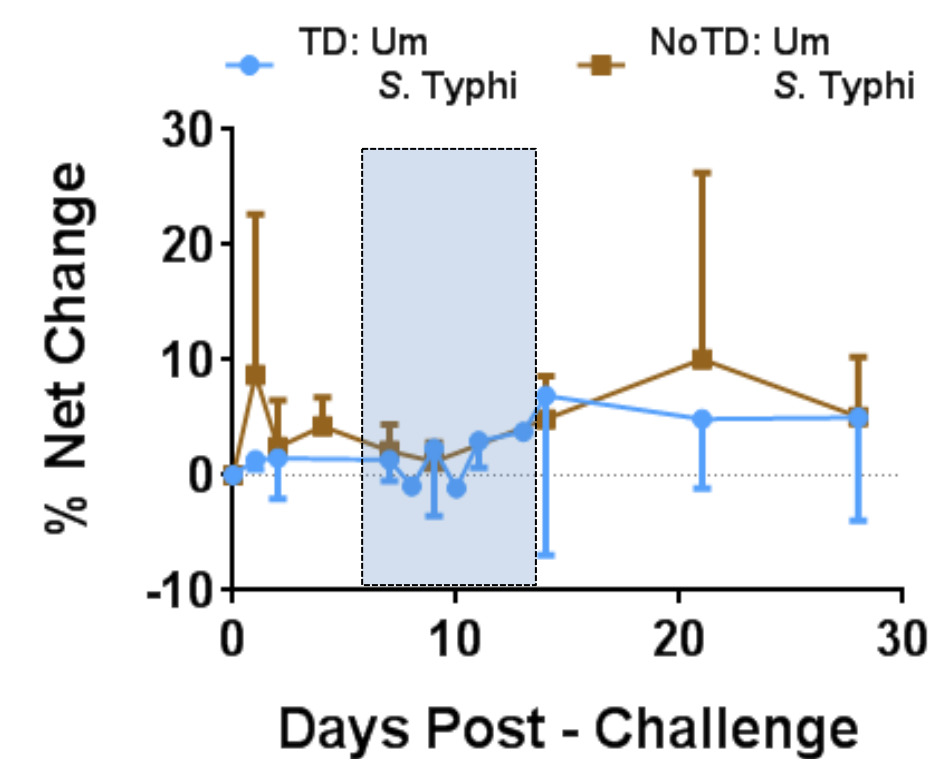**H**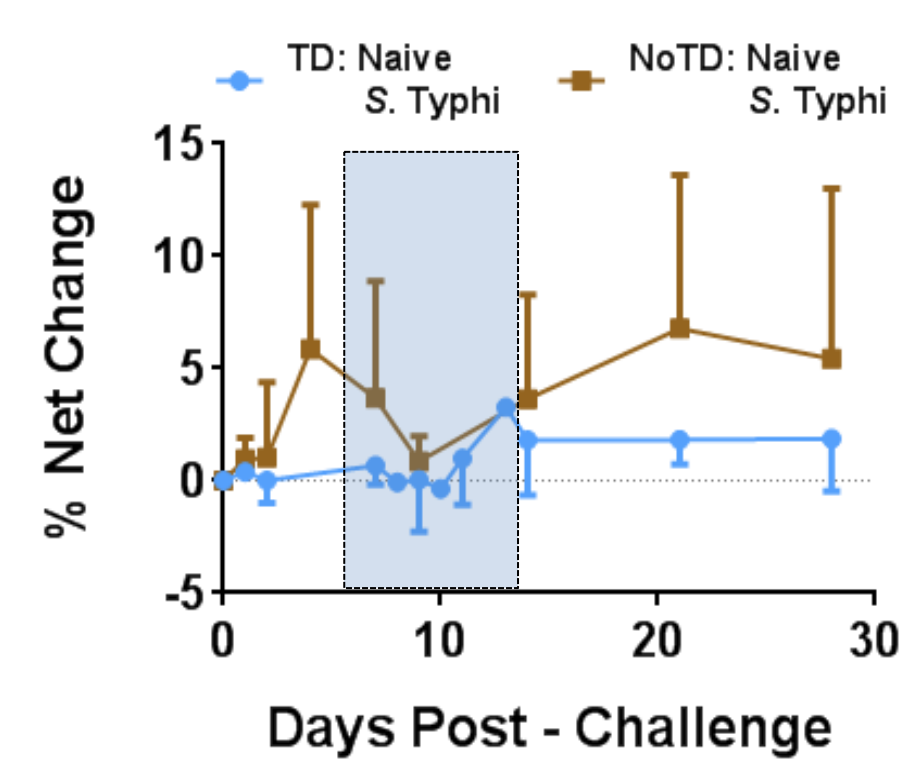

Supplement: S3 Fig — Panels A and B show examples of the changes in CD40 expression in naive cells (TD volunteer) and the time course of the net change (MdFI) of this molecule in naive cells. Panels C-H show the complete time courses of various other markers evaluated in the BM subsets. AroundTD is indicated by the blue rectangles with dotted lines in panels B-H. In the same panels TD and NoTD volunteers are indicated by the blue and brown symbols, respectively. Panels B-H display Mean ± SD. (PDF) [file pntd.0004766.s003.pdf]

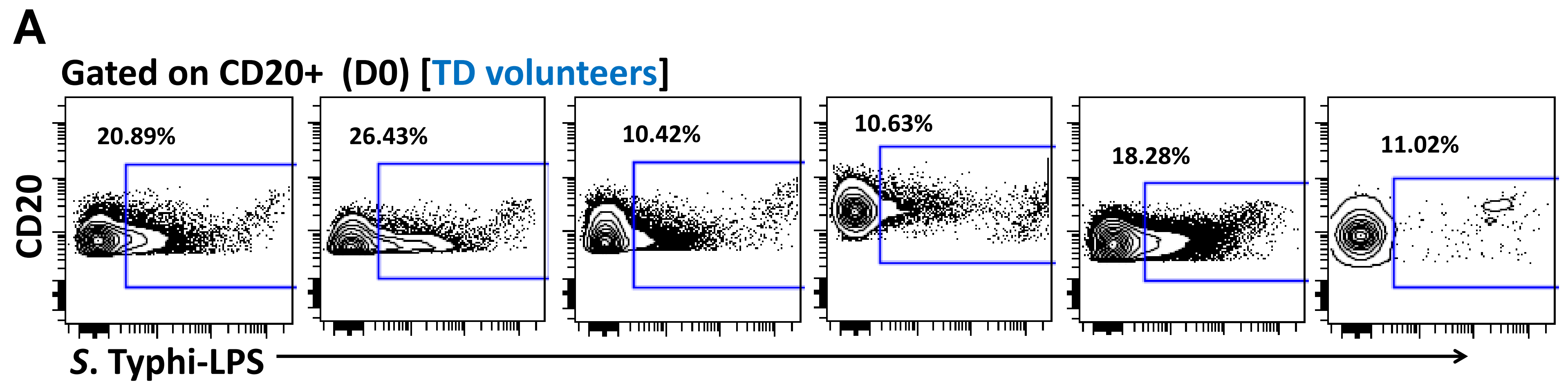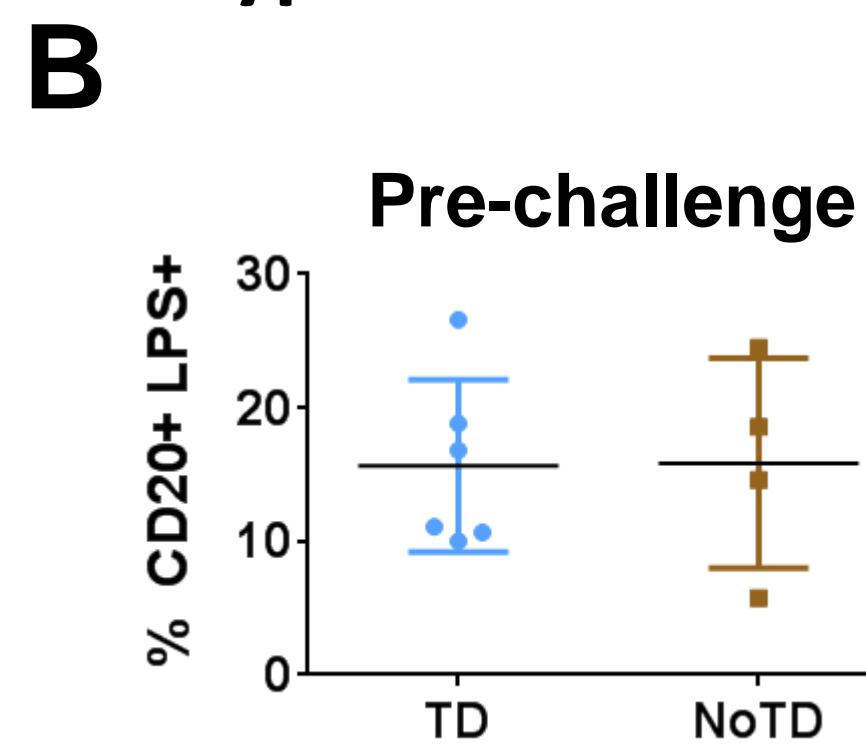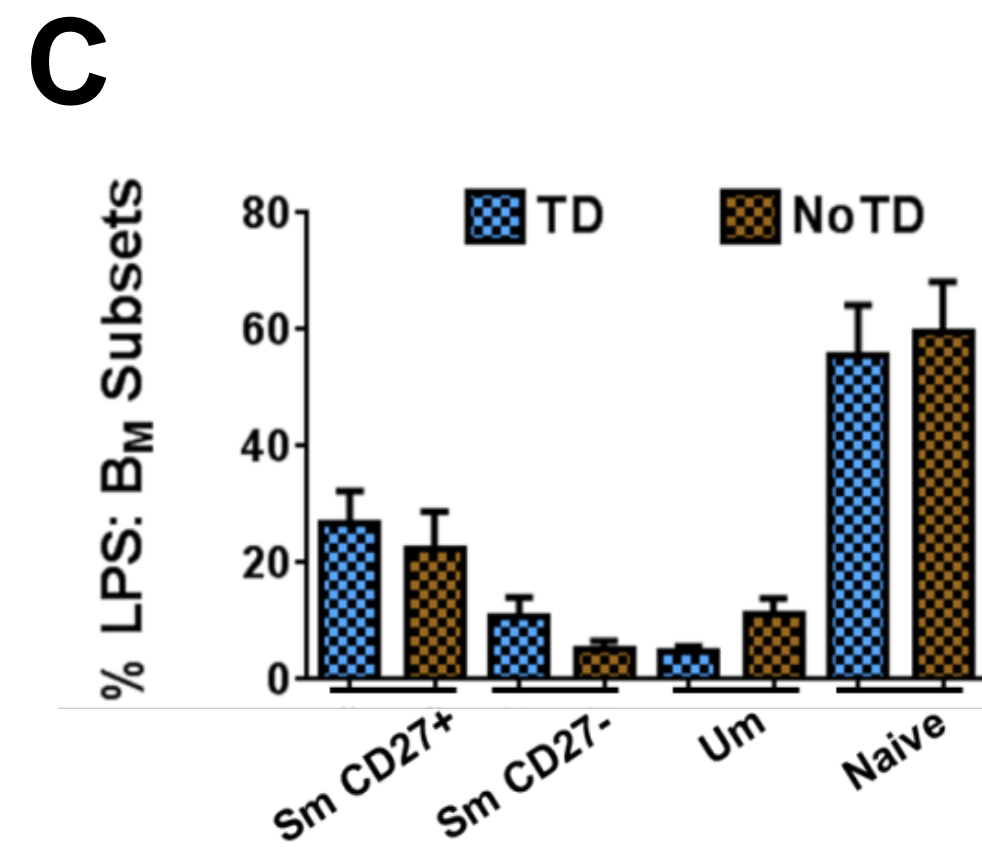

CD20+ LPS+: B<sub>M</sub> subsets ( # cells/ml)

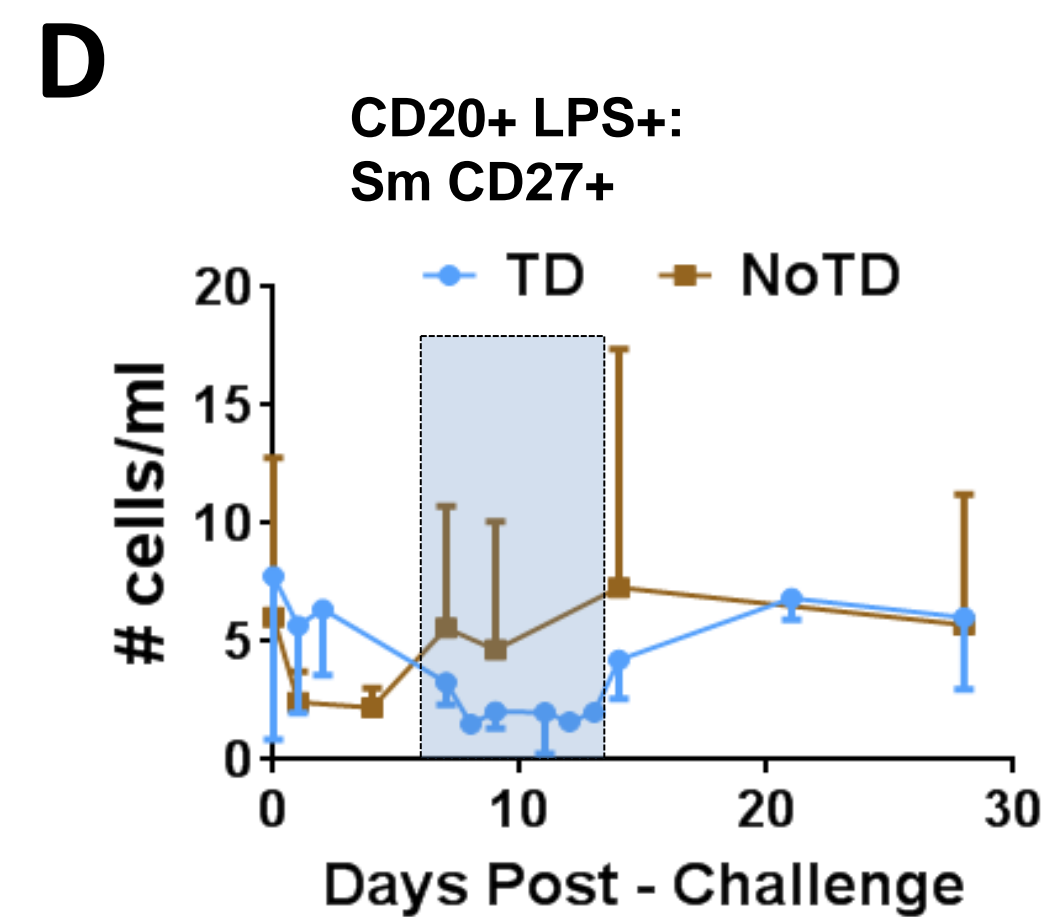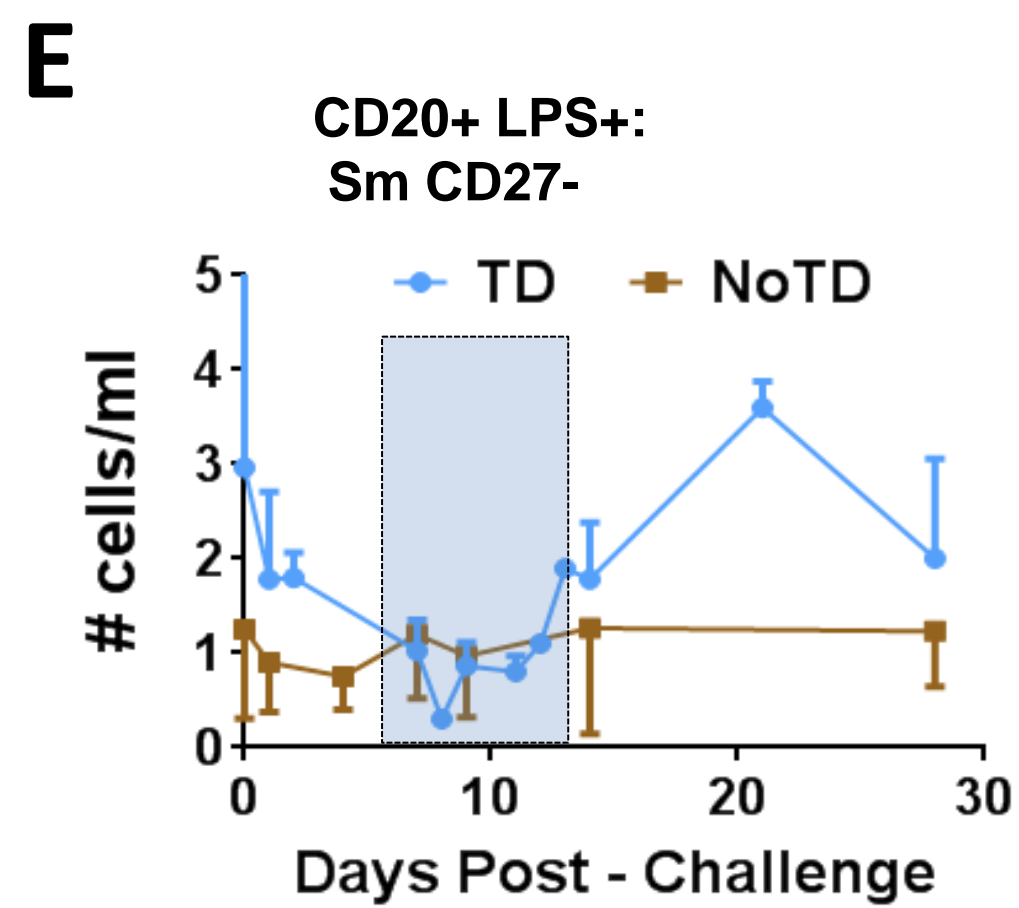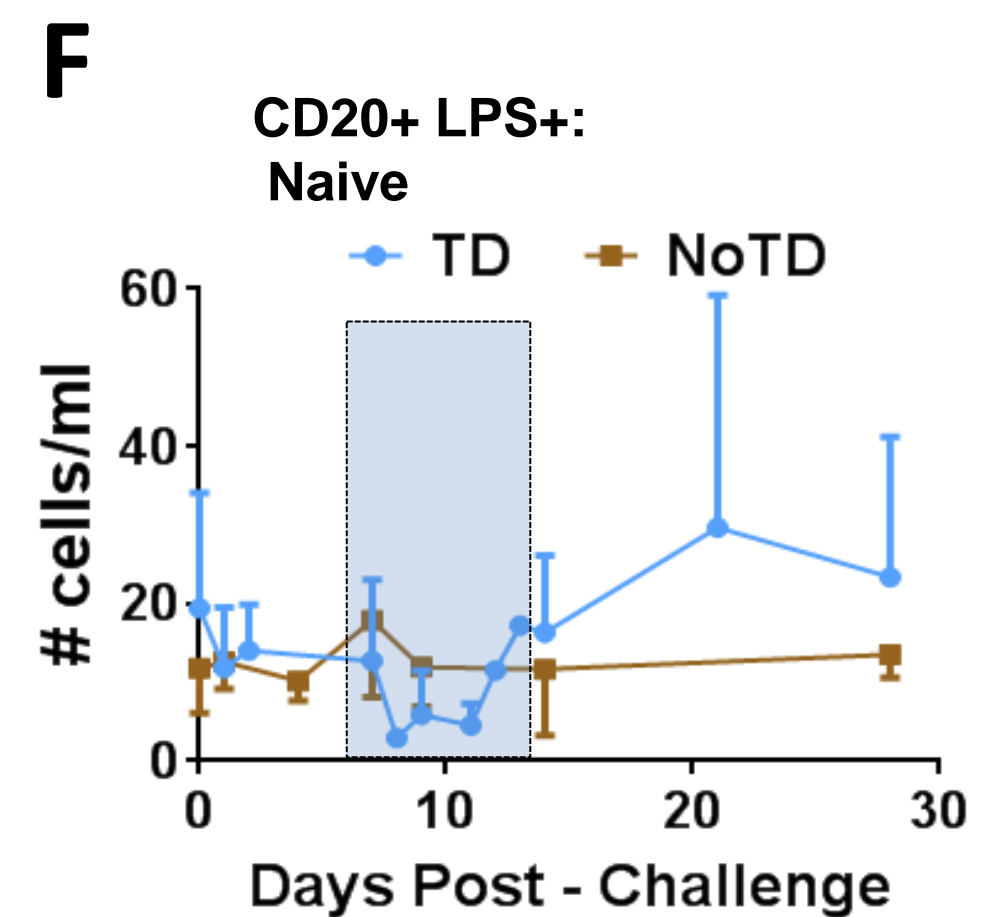

Supplement: S5 Fig — S. Typhi-specific B cells were identified and concomitantly stimulated using S. Typhi-LPS-nanoparticles. Panel A displays bi-exponential plots showing the gating of LPS+ cells within the CD20+ population. Volunteers evaluated in the TD group are shown and, as expected, a high non-specific binding of LPS-nanoparticles was identified due to the “sticky” nature of LPS. Panel B shows the percentage of CD20+ LPS+ B cells at day 0 (pre-challenge) in TD (blue symbols) and NoTD (brown symbols) groups and demonstrate that no differences between these groups existed. Panel C shows the percentage of BM cell subsets that are CD20+ LPS+ cells at day 0, demonstrating that no differences between TD (blue symbols) and NoTD (brown symbols) groups exist before challenge. Panels D-F display time courses of the frequency (cells/ml) of SmCD27+, Sm CD27- and Naïve cells. No differences between TD and NoTD groups were identified. Importantly, Um cells showed a significant difference (Fig 6) between TD and NoTD groups AroundTD. This is described in detail in the main body of the manuscript. In panels D-F AroundTD is indicated by the blue rectangles with dotted lines. In the same panels TD and NoTD volunteers are indicated by the blue and brown symbols, respectively. Panels D-F display Mean ± SD. (PDF) [file pntd.0004766.s005.pdf]
