# Supplementary material for: Oral Challenge with Wild-Type Salmonella Typhi Induces Distinct Changes in B Cell Subsets in Individuals Who Develop Typhoid Disease
Source: PLoS Negl Trop Dis. 2016 Jun 14;10(6):e0004766. doi: 10.1371/journal.pntd.0004766 (PMC4907489; doi:10.1371/journal.pntd.0004766)
Supplement: S4 Fig — The statistical analyses displayed were performed using a Mixed Effects Model (as described in materials and methods). Changes (increase or decrease) in a specific marker were evaluated in TD volunteers with respect to NoTD volunteers during the specific time frames indicated. Information found in the boxes include the time frame in which the changes were identified (e.g., AroundTD and/or AfterTD); how the marker was evaluated (e.g., % net change compared to day 0) and the P value from the Mixed Effects Model analysis. Significant data are shown in highlighted boxes (light green). These boxes also include the Fig numbers in which the data is presented in the manuscript. (PDF) [file pntd.0004766.s004.pdf]

Markers evaluated

B_M Populations	α4β7	IgA	CD40	CD21	*S* . Typhi
Sm CD27+	AroundTD (Net%; P*=0.57)	AroundTD (Net%; P=0.18) Fig S2F	AfterTD (MdFI; P=0.13)	AroundTD (MdFI; P=0.040) Fig 4B	AroundTD & AfterTD (Net%; P=0.96) Fig S2H
Sm CD27-	AroundTD (Net%; P=0.56)	AroundTD (Net%; P=0.44) Fig S2G	AfterTD (MdFI; P=0.42)	AroundTD (MdFI; P=0.0006) Fig 4E	AroundTD & AfterTD (Net%; P=0.77) Fig S2I
Um	AroundTD (Net%; P=0.58)	N/A	AroundTD & AfterTD (MdFI; P=0.043 & 0.034) Fig 4H-I	AroundTD (MdFI; P=0.15)	AroundTD & AfterTD (Net%; P=0.75) Fig S2J
Naïve	AroundTD (Net%; P=0.0067) Fig 5B	N/A	AfterTD (MdFI%; P=0.019) Fig 5F	AroundTD (MdFI; P=0.12)	AroundTD & AfterTD (Net%; P=0.26) Fig S2K

* P-values for differences between TD and NoTD [Mixed effects model]

N/A: Non-available
