# Supplementary material for: Oral Challenge with Wild-Type Salmonella Typhi Induces Distinct Changes in B Cell Subsets in Individuals Who Develop Typhoid Disease
Source: PLoS Negl Trop Dis. 2016 Jun 14;10(6):e0004766. doi: 10.1371/journal.pntd.0004766 (PMC4907489; doi:10.1371/journal.pntd.0004766)
Supplement: S6 Fig — S. Typhi-specific B cells were identified and concomitantly stimulated using S. Typhi-LPS-nanoparticles. Changes in the signaling profile induced by typhoid were evaluated in the various BM subsets. Panel A shows three examples of the changes in the percentage of cells phosphorylating Akt (pAkt) after challenge (day 0 vs. peak phosphorylation AroundTD) in TD volunteers. The gates were set up based on media only stimulation. The data were reported as the differential in phosphorylation between days post-challenge and day 0. Panels B, C, D and E show time courses of the net changes (percentages) in phosphorylation of signaling proteins showing significant differences between TD and NoTD volunteers AroundTD. These changes were present in SmCD27+, Sm CD27- and Naïve cells. In panels B-E AroundTD is indicated by the blue rectangles with dotted lines. In the same panels TD and NoTD volunteers are indicated by the blue and brown symbols, respectively. Panels B-E display Mean ± SD. Panel F shows a table summarizing the results of the signaling proteins evaluated in the different BM subsets. Information found in the boxes include the time frame in which the changes were identified (e.g., TD+0h to TD+96h); how the marker was evaluated (e.g., % net change compared to day 0) and the P value from the Mixed Effects Model analysis. Significant data are shown in highlighted boxes (light green). These boxes also include the Fig numbers in which the data is presented in the manuscript. (PDF) [file pntd.0004766.s006.pdf]

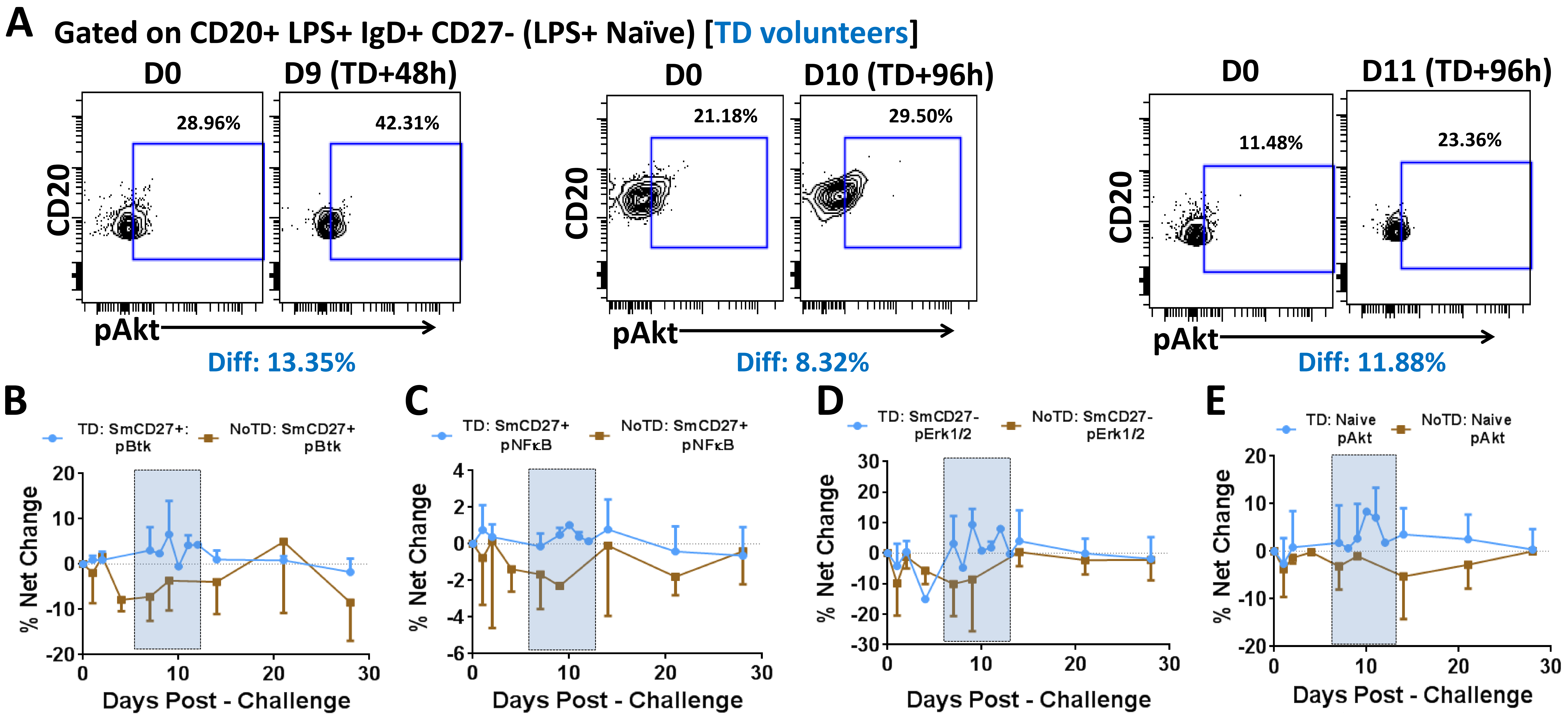

F

Markers evaluated

B$_M$ Populations	pAkt	pp38MAPK	pBtk	pNFkB	pErk1/2	pSyk
Sm CD27+	AroundTD (Net%; P*=0.61)	AroundTD (Net%; P=0.088)	AroundTD (Net%; P=0.024) Fig 7 B	AroundTD (Net%; P=0.011) Fig 7 E	AroundTD (Net%; P=0.12)	AroundTD (Net%; P=0.13)
Sm CD27-	AroundTD (Net%; P=0.93)	AroundTD (Net%; P=0.26)	AroundTD (Net%; P=0.21)	AroundTD (Net%; P=0.77)	AroundTD (Net%; P=0.037) Fig 7 H	AroundTD (Net%; P=0.11)
Um	AfterTD (Net%; P=0.50)	AroundTD (Net%; P=0.52)	AroundTD (Net%; P=0.35)	AroundTD (Net%; P=0.77)	AroundTD (Net%; P=0.17)	AroundTD (Net%; P=0.29)
Naïve	AroundTD (Net%; P=0.021) Fig 7 K	AroundTD (Net%; P=0.20)	AroundTD (Net%; P=0.48)	AroundTD (Net%; P=0.93)	AroundTD (Net%; P=0.31)	AroundTD (Net%; P=0.24)

* P-values for differences between TD and NoTD [Mixed effects model]
